# The adhesively-bonded glass brick system of the Qaammat Pavilion in Greenland: From research to realization

**DOI:** 10.1007/s44150-022-00031-2

**Published:** 2022-03-25

**Authors:** F. Oikonomopoulou, T. Bristogianni, M. van der Velden, K. Ikonomidis

**Affiliations:** 1 Department of Architectural Engineering + Technology, Faculty of Architecture and the Built Environment, Delft University of Technology, Delft, 2628 BL The Netherlands; 2 Konstantin Arkitekter, CVR: 41618418 Copenhagen, Denmark

**Keywords:** Cast glass, Glass bricks, Adhesive bonding, Glass blocks, Arctic architecture, Glass structure

## Abstract

An adhesively bonded, solid-glass brick pavilion has been designed by Konstantin Arkitekter as a landmark within the Aasivissuit – Nipisat UNESCO heritage in Greenland. The sculptural glass structure, measuring approximately 3.2 m in diameter × 2 m in height, faces a diverse set of engineering challenges compared to existing adhesively bonded glass brick structures. Placed in a remote location in the arctic circle, it has to withstand winter temperatures as low as -35 °C, and be built under a limited budget with the aid of the local population. Hence, key for the successful construction of the pavilion is finding an adhesive that satisfies the structural and aesthetic requirements of the project and simultaneously provides a simple and fast construction that spares the need for specialized building crew and sophisticated equipment, and is able to withstand the polar winter temperatures. Applicability and shear tests in (i) lab temperature conditions and (ii)) -5 °C lead to the final selection of: (a) *3M™ Scotch-Weld™ Polyurethane Adhesive DP610,* which has a higher shear strength capacity, 1 mm gap filling capacity and is clear in colour, for bonding the bottom rows of the pavilion where higher strength is required due to the reduced overlapping of the bricks; and of (b) DOWSIL Experimental Fast Curing Adhesive developed by Dow Silicones Belgium particularly for this project, with a satisfactory shear strength, 3 mm gap filling capacity and white colour for the rest of the pavilion; its considerably larger gap filling capacity facilitates the ease of assembly as it can accommodate within the joint thickness the anticipated ± 1.5 mm standard size deviations of the soda-lime cast glass solid bricks and the possible accumulated deviations during construction. The paper further describes the application of the adhesive, first on a small-scale prototype, and then on site, and presents the encountered engineering and logistical challenges during the construction of the pavilion in Greenland.

## Introduction

**Fig. 1:**
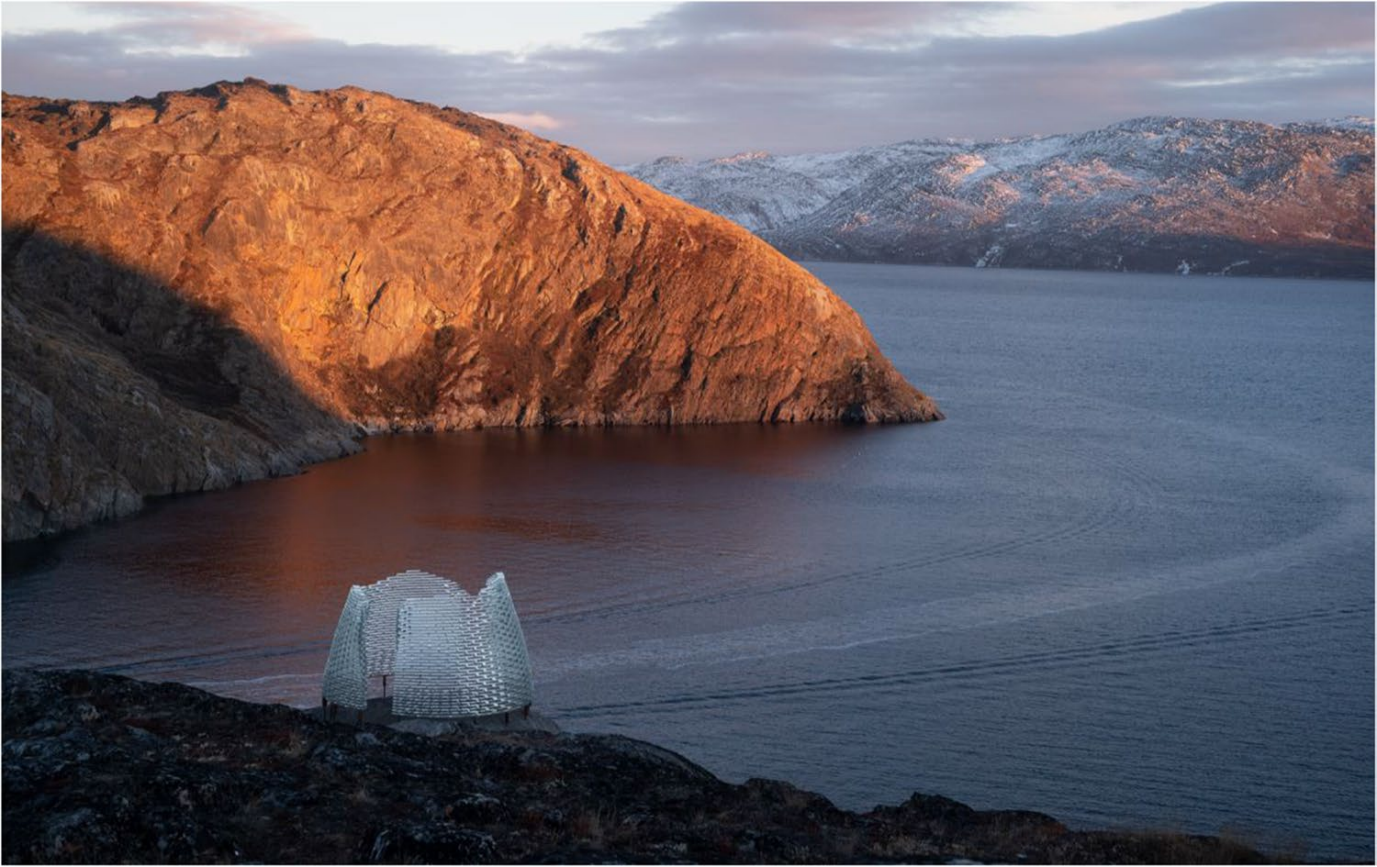
The completed pavilion. Photo credits: Julien Lanoo

An adhesively bonded, solid-glass brick pavilion has been designed by Konstantin Arkitekter as a landmark, sculptural structure, for a planned hiking route within the Aasivissuit – Nipisat UNESCO heritage in Greenland (Fig. 1). The *Qaammat Pavilion*, comprising 2 semi-circular units, and with total dimensions of circa 3.2 m in diameter and 2 m in height (excl. the elevated metal foundation), faces a diverse set of engineering challenges compared to existing adhesively bonded glass brick structures. In our case, the Qaammat Pavilion should be built under an extremely limited budget and with the aid of the local unskilled work force, calling for a simple bonding system. The pavilion’s location on top of a rocky hill, further supports this demand, as it implies a complicated access, an absence of electricity and of other commodities conventionally available in construction sites. Adding to this, the location is just north of the arctic circle, implying that the adhesively-bonded pavilion should be able to withstand ambient temperatures as low as -35 ˚C [1].^1^ Hence, in our case, key for the successful construction of the Qaammat Pavilion is finding an adhesive that satisfies the structural and aesthetical requirements of the project, can withstand the extreme winter temperatures of the polar climate and, equally importantly, can offer a simple, easy and fast assembly process that spares the need for a specialized building crew, sophisticated equipment and strictly regulated environmental conditions during its construction.

### Design

**Fig. 2:**
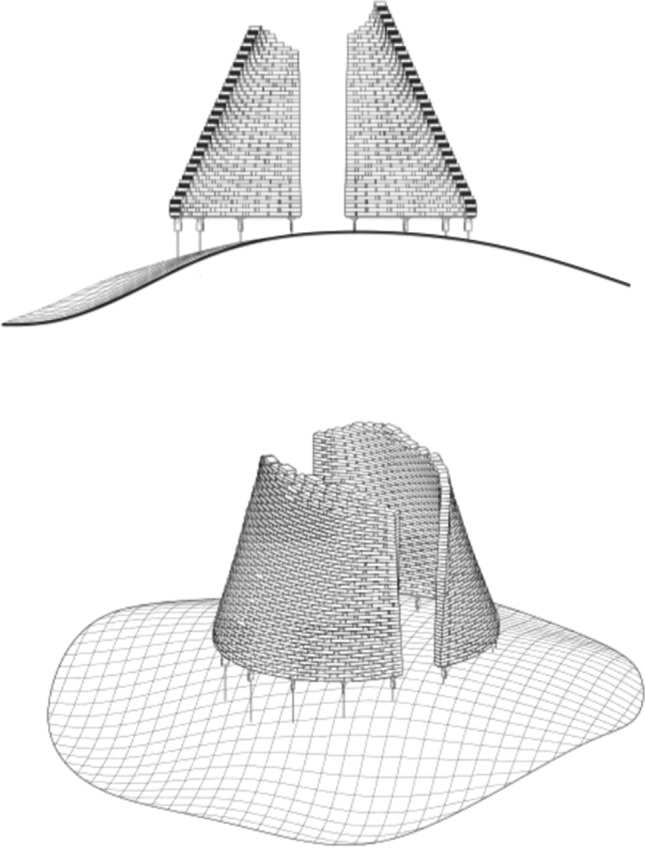
Final design of the 2 m high pavilion by Konstantin Arkitekter

**Fig. 3:**
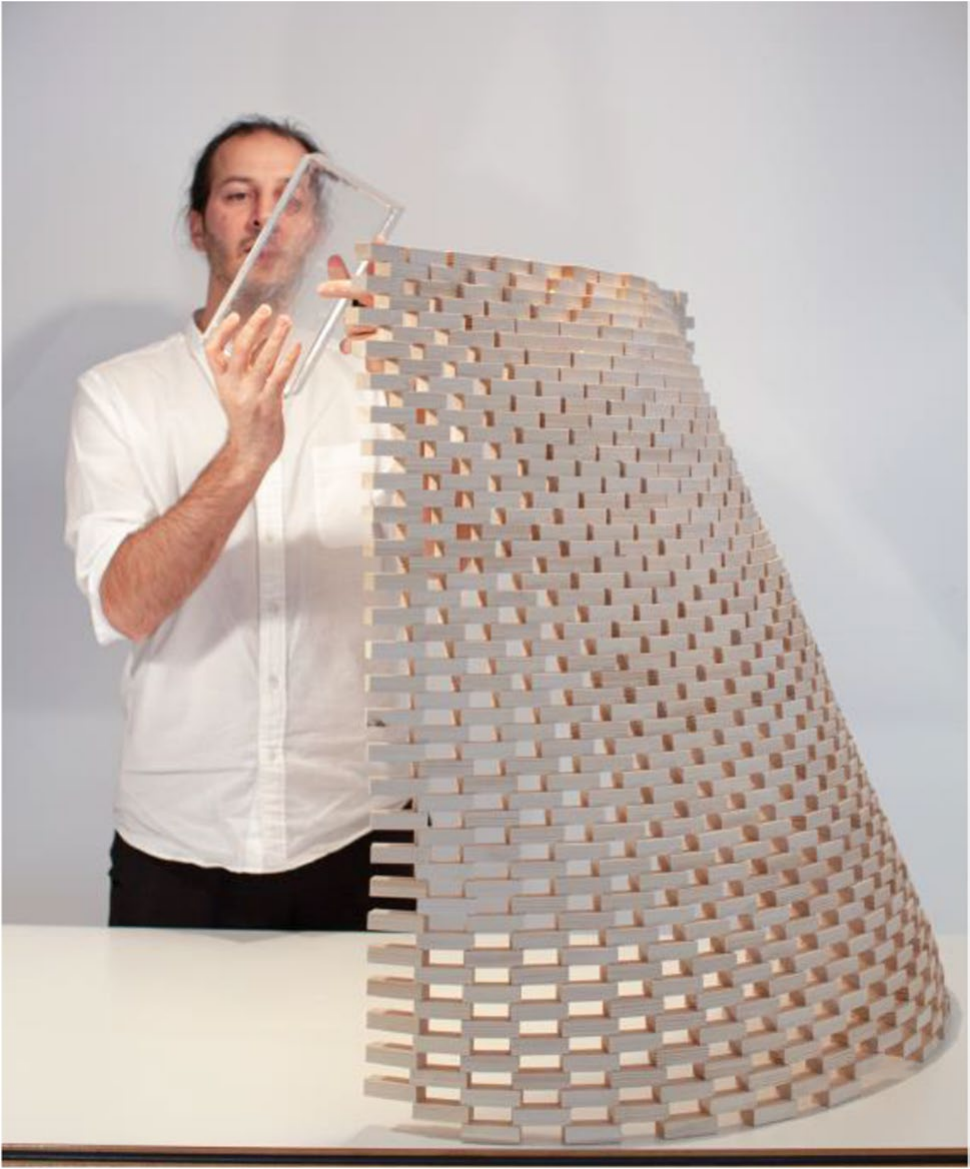
Architect K. Ikonomidis with a wooden mock-up of one of the pavilion’s walls

Following roughly a conical frustum in shape, the glass structure is circa 2 m in height (approx. 35 rows of glass blocks) by 3.2 m in diameter and consists of two distinct walls, each following approximately a semi-circle in plan and weighing approx. 2 tn. The two walls are slightly inclined towards the interior; thus, the distance among them is reduced towards the top of the construction (Fig. 2). The glass structure is designed to be perforated, in the sense that there are considerable gaps left between adjacent bricks (see Fig. 3). It comprises a total of approx. 1100 solid soda-lime cast glass bricks, produced by Wonderglass. Each brick measures 240 × 110 × 53 mm ± 1.5 mm and weighs circa 3.5 kg. A limited amount of smaller bricks (116 × 121 × 53 mm ± 1.5 mm) is also used at the two vertical edges of the two glass brick walls.

### Logistical challenges

The Qaammat Pavilion is placed at the outskirts of Sarfannguit, a small fishing settlement within the Aasivissuit – Nipisat UNESCO World Heritage Site, of approx. 100 inhabitants, located slightly north (coordinates: 66°53′50″N, 52°51′40″W) of the arctic circle (coordinate 66°30′ N). The polar climate imposes that the adhesively-bonded structure will have to withstand ambient temperatures as low as -35˚ C during the winter season.

**Fig. 4:**
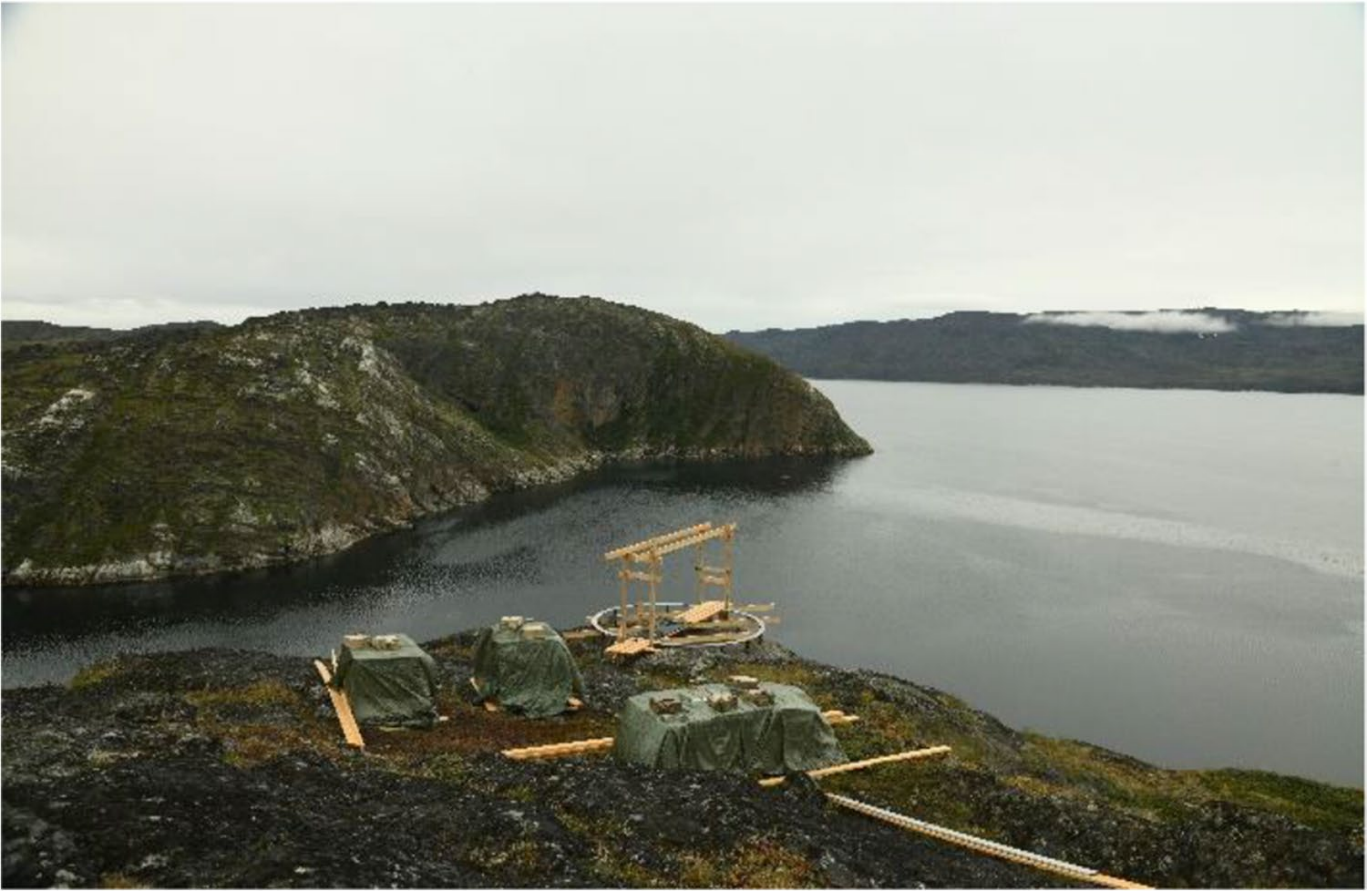
Location of the pavilion (outskirts of Sarfannguit)

**Fig. 5:**
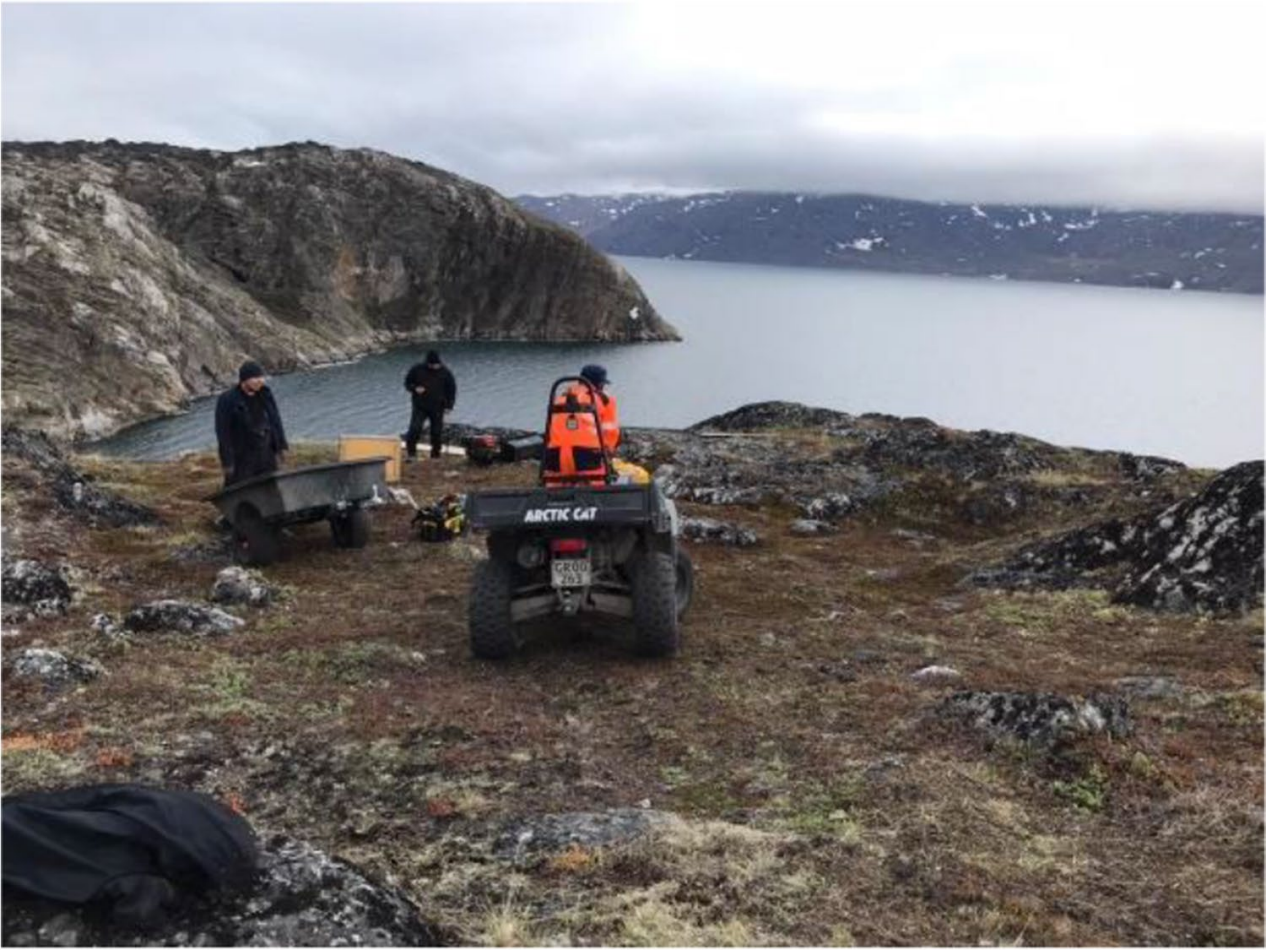
Access to the site is done via ATVs

The pavilion’s location in a rocky hill (Fig. 4) involves a complicated access (the site can only be accessed via ATVs/quad bikes, as shown in Fig. 5), absence of electricity and other common commodities in construction sites. Even within a tent installation, the site could only be heated by a gas heater; thus, regulated temperature and humidity conditions could not be guaranteed. Thus, adhesives that required electric dispensers for their pumping or strictly controlled environmental conditions during their application, should be avoided. Moreover, the bonding of the glass-block pavilion can only occur during the warmest months of July to September, when median day temperatures are the highest, at approx. 5–15˚C. Given that the R&D phase for the adhesively-bonded glass-block system of this project started in November 2020 and taking into account in the planning sufficient time for the manufacturing and shipping of the adhesive and relevant equipment to Greenland, the timeframe reserved for carrying out the R&D phase for the adhesively-bonded system was limited to a max. of 6 months.

The project had to be realized under an extremely limited budget, which only sufficed for the partial ordering and shipment of materials; thus, it’s realization relied heavily in volunteer work and material contributions. In specific, Wonderglass provided the bricks at cost price and Dow Silicones Belgium developed and sponsored the finally selected, customized DOWSIL Experimental Fast Curing adhesive. TU Delft’s contribution in the research & development and testing of the selected adhesives was also conducted voluntarily and gratis. As there was no budget allocated for appointing a structural engineering practice to perform the relevant structural analysis and verification, the final engineering of the structure relied instead on a set of performance criteria established based on knowledge from previous relevant adhesively-bonded cast glass block systems. The budget also did not suffice for recruiting a highly-skilled building crew. Hence, the Qaammat Pavilion would be built by the architect, with the aid of the TU Delft researchers and of a few local residents. The lack of technical means and of a trained, experienced building crew deemed necessary that the selected adhesive should allow for a relatively simple and fast assembly process. The selected adhesive should have sufficient gap filling capacity to accommodate possible discrepancies in the size and surface quality of the bricks during construction, similarly to the function of a mortar in standard brickwork. This is fundamental not only for an easy assembly, but also for preventing the post-processing of the finishing surface of the bricks (to flatten them) which could lead to a significant increase in the production cost of the bricks [2].

## Previous relevant examples

Table 1 summarizes the main characteristics of relevant previous examples. In typical structural float glass applications, the developed adhesive solutions are engineered either for continuous linear connections (i.e. bonding along the length of a glass sheet) or for localized surface connections. The former call for fundamentally different adhesive solutions, designed for narrow joints, while the latter typically involve adhesives that exhibit a virtually zero layer thickness, enabled by the minimal allowable deviations in the thickness of float glass sheets (± 0.3 mm for glass up to 12 mm thickness and ± 0.5 mm for 15 mm thick glass according to [3]). This renders conventional adhesive solutions unsuitable for the discussed cast glass system, where the aim is to compensate for dimensional discrepancies within the bond thickness.

**Table 1:** Summary of main characteristics of relevant case-studies, derived from [4–8]

Case study	Crystal-Houses	Atocha Memorial	Qwalala Sculpture	Glass Sphinx	Glass Angel
Glass type	Cast glass blocks	Cast glass blocks	Cast glass blocks	Glass sheets	Glass sheets
shape	Flat, rectangular façade	Cylindrical envelope	Curved wall	Free-gate like shape	Angel statue
Height	10 m	11 m	2.4 m	6 m	3.5 m
Brick modulus	210 × 105 × 65 (± 0.25) mm	300 × 200 × 70 (± 1) mm	160 × 160 × 320 mm (deviations unknown)	600 layers × 10 mm thick glass	370 layers × 8 mm thick glass
Total weight	~ 40 tn	~ 130 tn	~ 69 tn	~ 94 tn	unknown
Prioritized adhesive selection criteria	- high bond strength - high creep resistance - high transparency	- high bond strength - high creep resistance - high transparency - accommodation of brick size deviations	- constructability - accommodate thermal movements - visual result (low shear stresses and increased stiffness due to optimized geometry)	- constructability (resulting stresses were very low)	- constructability
Selected adhesive	Delo Photobond 4468 UV-curing acrylate	Customized UV-curing acrylate	DOWSIL™ 993 Structural silicone	AFTC Silver Tape (100% Acrylic Foam)	3M™ VHB Tape
Thickness	0.25 mm	2.5 mm	7 mm	0.25 mm	unknown
Bond Strength	high	high	satisfactory (low)	unknown	unknown
Color	clear	clear	white	unknown	clear
Service T	-40 °C to 120 °C	Unknown (experimentally tested for -20 °C to 80 °C)	-50 °C to 150 °C	unknown	unknown
Construction challenges during installation	- Extreme accuracy required - Controlled environ. Conditions - highly controlled bonding process - CNC post-processing of bricks required	- Accumulated constr. tolerances - Controlled environ. Conditions - highly controlled bonding process - use of press-moulds for higher accuracy of glass blocks	- Proper mixing of the adhesive	- Controlled environ. conditions - Glass sheets should be placed absolutely horizontal and in-plane with each other - Minor cracking occurred due to uneven settlement during installation and once the adhesive tape settled	unknown

Perhaps the most closely linked float glass examples are the horizontally-layered glass sheet sculptures of the 6 m high Glass Sphinx (NL) and the 3 m high Glass Angel (NL). Both cases opted out of a conventional adhesive solution, illustrating the fact that structural glass adhesives are yet to be developed for bonding considerably large surfaces of glass in a stacked configuration.^2^ Instead a 3M™ VHB adhesive tape was used for the Glass Angel [8] in order to facilitate construction and a 0.25 mm thick AFTC Silver Tape (acrylate tape) for the assembly of the Glass Sphinx [7], which could still accommodate within its adhesive layer thickness the deviations in the thickness of the float glass panels. Even with the high accuracy level of float glass thickness and the use of a thin tape, an accumulated height offset of 4 cm was recorded during the construction of the Sphinx [9]; illustrating well the fact that considerable tolerances can easily arise during construction.

Up to now, there are only a few realized adhesively-bonded glass structures in external applications. The most characteristic ones are the self-supporting envelopes of the Atocha Memorial (ES) and the Crystal Houses façade (NL) and the sculptural structure Qwalala (IT). The developed adhesive systems for both the Atocha Memorial and the Crystal Houses façade, built in locations with moderate climates, focused primarily on the structural and visual performance of the selected adhesive, leading in both cases to the selection of clear, UV-curing acrylates of high-strength, yet of a limited application thickness. In particular, in the 11 m high Atocha Memorial, the cylindrical shape of the structure in combination with intensive testing and the use of mould-pressed borosilicate glass bricks of higher dimensional accuracy, enabled the use of a custom-developed UV-curing acrylate that can be applied in a layer thickness up to 2.5 mm and can accommodate the size deviations (± 1 mm) of the glass bricks [10]. On the other hand, the flat geometry of the 10 m high Crystal Houses glass-block façade imposed the use of Delo Photobond 4468, with an optimum layer thickness of 0.25 mm [11], calling for the post-processing of the soda-lime glass blocks to a matching flatness and height accuracy (± 0.25 mm). In this case as well, a series of experimental tests and prototypes, carried out over a period of 18 months, were necessary in order to validate the structural and visual performance of the assembly. The inability of acrylic adhesives to accommodate accumulated construction tolerances within their thickness, combined with the architectural prerequisite of high visual performance, led to most of the involved engineering challenges in these projects. In both projects, strict tolerances had to be met not only per construction layer but also for the entire structure [2], imposing the need for a highly skilled crew and a meticulous construction, further enforced by the controlled environmental conditions (temperature, humidity and UV-radiation) necessary for applying acrylic adhesives. Both envelopes were sealed using a more flexible bonding media to prevent dirt, water and humidity from entering in the joints.

The 2.4 m high Qwalala sculpture by Artist Pae White, is perhaps the closest realized example to the discussed case-study. A shape optimization study conducted for this sculpture facilitated the use of a flexible adhesive instead by significantly reducing the relevant shear (max. shear stresses were found to be 0.18 MPa) and normal stresses within the adhesive layer and reinforcing the rigidity of the structure [5]. In this case, Dowsil™ 993 Structural Glazing Sealant, a two-component structural silicone of white colour, high UV-resistance, 0.95 MPa tensile strength and 7 mm layer thickness, was applied in blobs for bonding the 3000 glass bricks of the curved glass wall, each weighing 23 kg [6]. There was no additional sealant used in this project.

## Adhesive prerequisites and preselection

### Establishment of performance criteria

In our case, the pavilion had to be built under an extremely limited budget, withstand arctic temperatures and be constructed by an amateur building crew. Accordingly, the ease-of-assembly of the construction and need for stable properties over a wide temperature range proved to be more critical aspects for the adhesive selection than obtaining maximized strength and acquiring a fully transparent, flawless, appearance. The focus was placed primarily in finding a structural adhesive that functions similarly to a mortar in traditional brickwork in order to facilitate assembly: the adhesive should provide sufficient strength and at the same time absorb, within its thickness, the intolerances in size of the bricks and of the entire construction and allow for a fast and simple assembly. Subsequently, the prioritized performance criteria for the adhesive selection for the Qaammat pavilion, are fundamentally different to the ones followed by the previous realized examples of adhesively-bonded glass brick envelopes, namely the Crystal Houses façade [4] and the Atocha Memorial [10],and are more similar to the bonding solution followed at the Qwalala Sculpture (see Table 1), although in our case all joints should be sealed afterwards to prevent water/frost and dirt from entering.

**Fig. 6:**
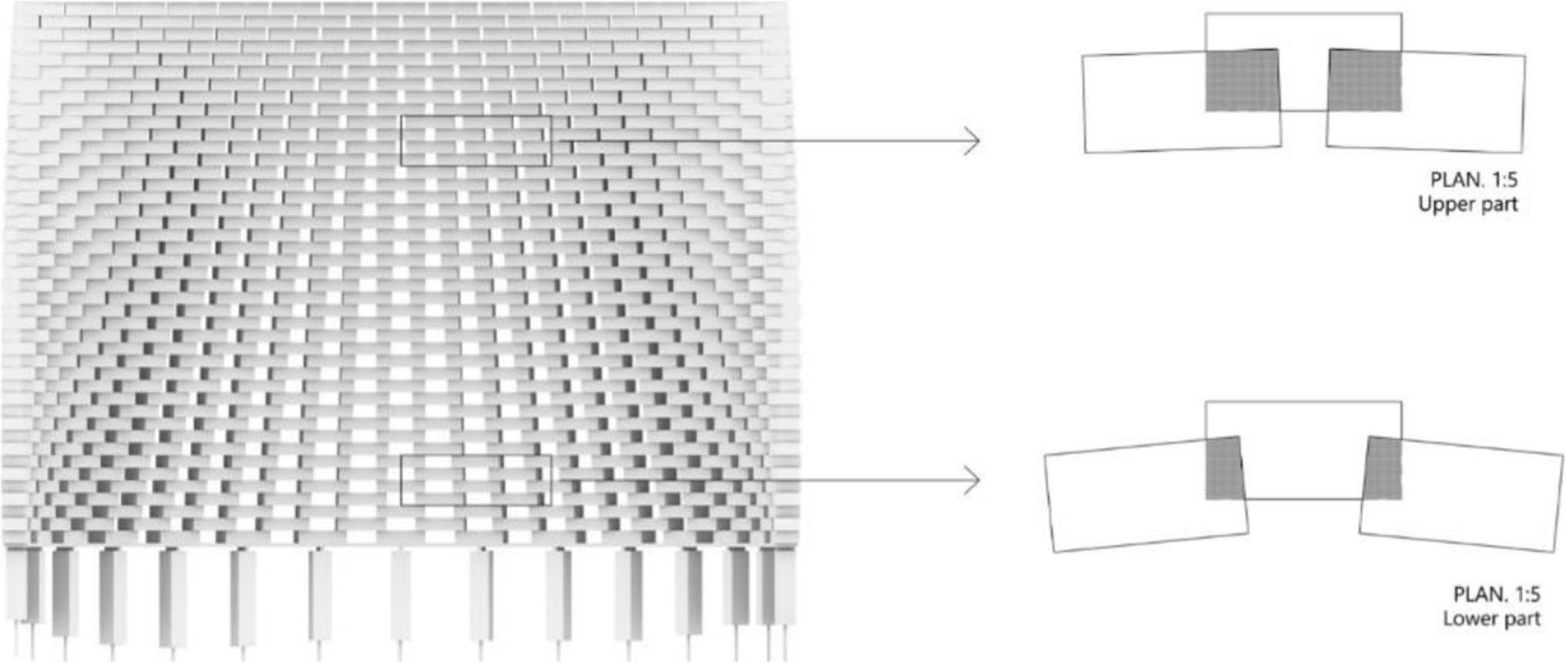
View of one of the two walls by K. Ikonomidis showing the larger overlap between bricks in the middle and top part of the construction and the smaller overlap in the lower part of the structure. It should be noted that in the final construction, the bricks are considerably less protruding than in the illustration

In terms of structural performance, due to the lack of a structural analysis model, several assumptions had to be made for selecting a suitable adhesive. Owing to the high degree of perforation of the structure that reduces wind pressure due to lateral wind gusts, tensile resistance properties were not considered crucial. A shear strength ≥ 1 MPa was established as desirable at a wide temperature range, based on the previously realized examples. However, due to the lesser overlap of the blocks (resulting to a reduced bonding cross-section) and the bending stresses occurring due to the inclined cantilevering of the two walls at the lower part of the glass structure (Fig. 6), an adhesive of a higher strength would be more favourable at this zone. A high creep resistance is not considered critical for this structure: considering the total dimensions and weight of each wall (circa 2 tn) and assuming an even load distribution, the expected pre-compression due to the own weight of the structure at a brick in the first row of the pavilion with 20% of its total surface bonded is < 0.22 MPa. The chosen adhesive should present stable properties at a wide temperature range, particularly against ambient temperatures as low as -35 ˚C, recorded in this location.^3^

Ease-of-assembly was equally critical: the thickness of the adhesive should be able to accommodate the manufacturing tolerances of the glass bricks (± 1.5 mm) and further size discrepancies which may occur during construction, thus a 3 mm gap-fill capacity was considered essential^4^; this was also desired to compensate for movements due to thermal differentials of the glass bricks. Moreover, the selected adhesive should allow for a fast fixing and curing time, which were set at < 30 min and < 24 h respectively. A quick fixing time was important for preventing the overflow of the adhesive and accidental movement (sliding) of the blocks but also for enabling a relatively quick construction, essential due to the short Greenlandic summer: the pavilion should be built within a few weeks, thus, the adhesive should set quickly enough to allow for the built-up of several rows (3–4) in one day. Lastly, due to the lack of electricity and of other common commodities in the specific location, it was essential that the construction could be realized without the need of strictly controlled environmental conditions (i.e. regulated humidity and temperature levels). In terms of visual performance, although a fully-transparent adhesive was the most desirable, adhesives of a white or light grey colour were also acceptable as a solution by the architect.

In specific, the following key factors, in terms of structural performance, visual result and ease of assembly, have been established for the adhesive selection:

1. **Structural performance:**
  Shear strength ≥ 1 MPa and adequate tensile strength – a higher strength is more favourable at the lower levels of the construction
  Stable mechanical properties over a wide temperature range, as low as -35^o^ C
  ability to equalize stresses (prevention of stress concentrations, e.g. due to locally insufficient contact with the glass substrate or due to voids within the adhesive layer)
  ability to accommodate movements due to thermal expansion to prevent thermal breakage
2. **Visual performance:**
  Transparent, translucent or white/light grey in colour, in order to maintain a high level of transparency
  Very good resistance to UV-radiation
  can be homogeneously spread (prevention of overflow and of bubbles, gaps and dendritic patterns)
3. **Ease of assembly:**
  fast fixing (< 30 min) & curing time (< 48 h)
   > 3 mm gap filling ability
  no emissions of noxious or poisonous chemicals
  no need for strictly regulated environmental conditions during construction

### Choice of most suitable adhesive family

**Fig. 7:**
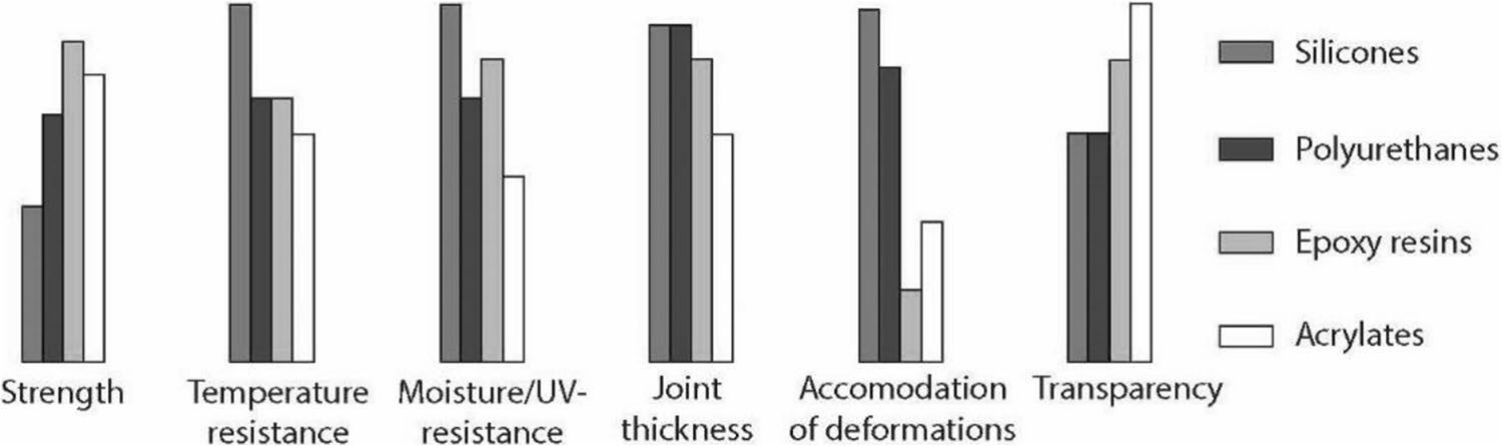
Qualitative comparison of various adhesive systems derived from [12]. In our case the temperature resistance and joint thickness were considered the most critical aspects to ensure a simple, fast construction that can withstand the arctic winters of Greenland

The arctic climatic conditions of Greenland pointed out towards two-component flexible adhesives, from the polyurethane and silicone-modified families as the most suitable adhesive family. These families are known for their excellent stability of mechanical properties over a broad temperature range (see Fig. 7). Moreover, such flexible adhesives typically present tensile and shear strength > 1 MPa and a bond thickness sufficient for accommodating dimensional tolerances and for equalizing stresses [12]. The strength of the Si–O bond provides silicones with high UV-resistance and allows them to be extruded even in temperatures < 0˚C [13]. Equally importantly, the chemical hardening process of such adhesives is less influenced by external climate conditions, allowing for construction conditions that do not require strictly regulated levels of temperature and humidity, a necessity in this case. In particular, silicone sealants have been previously successfully used in bonding and sealing applications in arctic climatic conditions, such as in the Princess Elizabeth Research Station in Antarctica [14], but also for the bonding of a similar cast glass structure, i.e. the Qwalala sculpture in Italy [5].

In specific, one-component moisture/heat activated adhesives were quickly discarded as an option due to their physical hardening process. This type of cure chemistry requires a favourable water vapour pressure in the atmosphere, which is a function of both temperature and humidity; which in our case could not be fully regulated. Moreover, the curing of such adhesives takes place from outside to inside at a relatively slow rate (of a few mm per day), rendering them unsuitable for wide joints: as the adhesive solidifies and thus, shrinks on its surface, tensile forces develop that can be sufficient to tear the still soft, uncured adhesive at the interior of the bond.

Epoxies and acrylates, despite presenting the highest strength among the adhesive families typically used in structural glass applications (see Fig. 7), including in the construction of both the Crystal Houses façade [15] and the Atocha Memorial [10], were in principle considered unsuitable for this case-study, due to their reduced application thickness/gap-filling property (typically between 0.1—0.5 mm) that does not allow them to accommodate construction tolerances[12].^5^ Moreover, their application calls for thoroughly controlled environmental conditions during construction, which could not be secured at the pavilion’s location.

Furthermore, the uneven surface of the cast glass bricks hindered the application of double-sided transparent tapes, previously used in float glass layered sculptures (see Table 1), as they are in principle unable to accommodate the dimensional tolerances of the cast blocks and contraction and expansion movements expected due to the extreme climatic conditions.

Cement-based mortars used for hollow glass bricks, although initially considered, were soon eliminated as an option as well, as, besides not meeting the visual requirements due to their darker colouring, they further require a rougher surface to achieve a good bond than the smooth surface of solid glass blocks. Indeed previous shear experiments at TU Delft have pointed out that even with the application of a primer, such mortars still do not tend to properly bond to the glossy surface of solid glass blocks and can easily lead to adhesive failure at low strength values [16]. Experimental work on solid glass blocks bonded with a selection of mortars by [17] further confirmed the adhesion collapse mode and indicated a shear strength considerably less than 1 MPa on glass blocks with a smooth finishing surface.^6^ Tile adhesives were also discarded as a choice, due to the fact that they are engineered primarily for indoor applications and are in principle not suitable for the low temperatures of Greenland.

### Adhesive preselection

Based on all the above, on market availability and upon consultation with the Institute of Building Construction of TU Dresden, Dow Silicones Belgium and Siko B.V, a selection of suitable transparent and white two-component adhesives in the polyurethane and silicone-modified families were selected for further exploration. A two-component adhesive from the acrylate family was also selected as it fulfilled the established criteria and presents high bonding strength.

In specific, the following four adhesives were selected for further investigation: (A) 3 M™ Scotch-Weld™ Polyurethane Adhesive DP610, (B) Teroson MS 9399, (C) Experimental Fast Adhesive by Dow Inc., (D) Siko Clearbond. More specifically, adhesives A, B and D are available in the market, while adhesive C was specially formulated by DOW Silicones Belgium for the purposes of this project, as none of the commercially available bonding solutions from Dow’s High Performance Building Solutions range checked all of the project’s requirements: This adhesive has been formulated by DOW using a 4:1 Vol. mixing ratio with the aim to reach a lap shear strength of ~ 1 MPa in 1 h versus the standard mixing ratio of 100:14 weight that requires 24 h to reach the same strength. The snap time is reduced to 4—6 min and the time to handle strength to approx. 24 h. Moreover, DOW has removed the colouring pigment of the reacting component in order to achieve a final white colour instead of dark grey [13].^7^ The properties of the selected adhesives, as provided by the manufacturers, can be found in Table 2 below.

**Table 2:** Characteristic properties of the selected adhesives as provided by the manufacturers

adhesive	A	B	C	D
	**3M™ DP 610**	**Teroson MS 9399**	Dowsil Experimental Fast Adhesive by Dow*	**Siko Clearbond**
**type of adhesive**	2-component polyurethane adhesive	modified silicone polymer	two-part alkoxy RTV silicone	2-component methyl methacrylate
**chemical base**	urethane	silicone	silicone	acrylate
**colour**	clear	white	white	clear
**application thickness**	~ 1–2 mm	~ 2 mm	~ 2–3 mm	~ 2 mm
**setting time [at 24 °C]**	~ 10 min	~ 20 min	~ 20 min	~ 2 min
**time to handling strength [at 24 °C]**	~ 2 h	~ 2 h	~ 24 h	~ 4 min
**viscosity**	fluid	pasty, thixotropic	pasty, thixotropic	fluid
**lap shear strength (to glass)**	23 MPa at + 23 °C 34 MPa at -40 °C	2 MPa (to steel)	≥ 1.0 MPa	15–21 MPa
**tensile strength**	unknown	3 MPa	> 2 MPa	13–17 MPa
**elongation at break**	unknown	130%	250%	unknown
**UV-resistance**	excellent	excellent	excellent	excellent
**Service temperature**	-50 °C to 80 °C	-40 °C to 100 °C	-50 °C to 180 °C	-50 °C to 120 °C

*Data as stated in the datasheet by [18] for adhesive A, [19] for adhesive B. The data for adhesive C are provided directly by DOW Silicones Belgium. Testing at DOW suggested that a 2 mm thick lap shear between glass and stainless steel develops 1.2 MPa strength after 1 h and 1.4 MPa after 7 days of cure. Tensile strength exceeds 2 MPa for dumbbell testing at 2 mm thickness [13]. The data for adhesive D are based on personal correspondence with supplier Siko B.V

## Adhesive Testing and final selection

### Applicability tests

Initially, adhesive application tests, i.e. bonding two glass bricks together by dispensing adhesive in an “X” shape in the middle of the bottom block’s surface, were performed in order to further understand and evaluate the speed of reaction and strength development, evaluate the ease-of-assembly and get acquainted with the necessary equipment to dispense each adhesive. The applicability tests led to the further reduction of the candidates to three: Adhesive D (Siko Clearbond) was discarded due to practical considerations linked to its application for this specific case-study. Its fast setting (2 min) and handling time (4 min) was deemed marginal for ensuring the proper application of the adhesive and bonding of the glass bricks by an unskilled building crew; hence, it was determined to discard this adhesive as an option (see Table 3).

**Table 3:** Main empirical findings of the applicability tests

Adhesive	Ease-of-application	Ease-of-cleaning overflow	Spacer thickness	colour	Cartridge volume and ratio	Dispenser
A. 3M™ DP 610	Very easy to apply	Becomes sticky and should be scrapped; remaining traces can be cleaned with isopropanol	1 mm	clear	48.5 ml 1:1	Manual
B. Teroson MS 9399	Requires some pressure to be extruded	Does not overflow easily; can be easily cleaned	1.5 – 2 mm	white	50 ml 1:1	Manual
C. Dowsil Exper. Fast Curing Adhesive	Requires high pressure to be extruded	Easy to clean overflow	2–3 mm	white	400 ml 4:1	Battery or pneumatic -driven
D. Siko Clearbond	Easy to apply; yet sets too quickly to be properly handled by non-skilled building crew	Hardens very quickly; hard to clean	N/A	clear	50 ml 1:1	Manual

The remaining three adhesives proved to be relatively easily applied and handled. In specific adhesives (A) and (B) come in small dual-tube cartridges (circa 50 ml) and can be easily extruded by a manual 50 ml 1:1 dispenser^8^; whereas (C), due to the 4:1 ratio, high viscosity and packaging in 400 ml cartridges, required the use of a pneumatic or battery-driven dispenser^9^; extrusion of this adhesive with a manual dispenser is also possible but requires considerable manual force.^10^

**Fig. 8:**
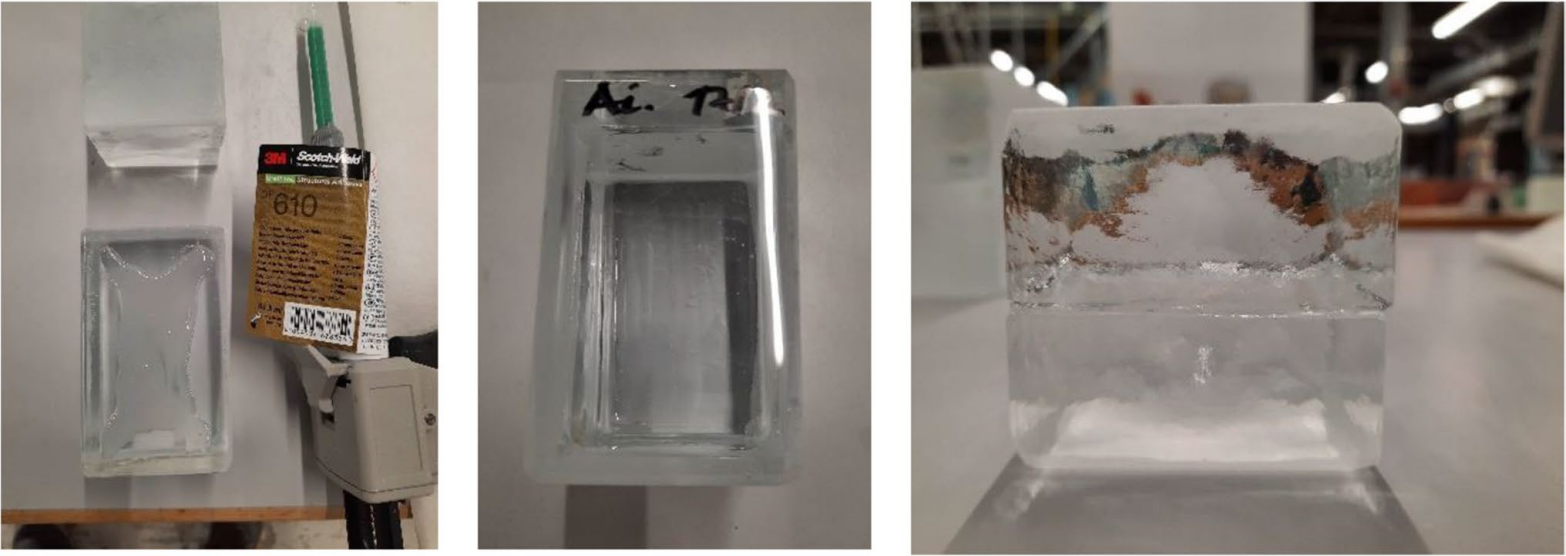
Application of 3M™ DP610 adhesive using a manual dispenser. The adhesive has a milky appearance once dispersed (left), yet turns entirely clear once bonding is established (middle). The adhesive spreads easily and homogeneously and only a few miniscule entrapped air bubbles are observed from top view. The clearness of the adhesive and the distorting side surface of the glass bricks makes the adhesive layer invisible from side view (right)

**Fig. 9:**
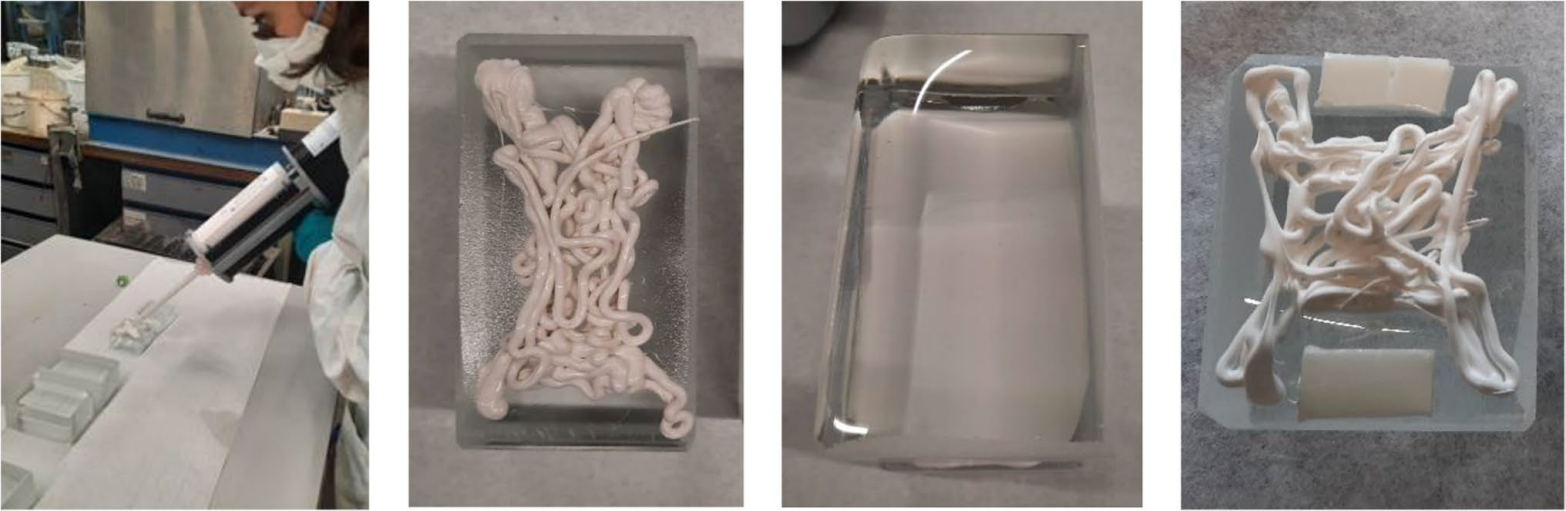
DOWSIL™ Experimental Fast Curing Adhesive application trials. The silicone is applied via a pneumatic gun (left). Initially the silicone is dispensed in an “X” shape (left middle), leading to an even spread with a minimum pressure from the brick above (right middle) but a difficult to control thickness of the silicone. Use of spacer tape is deemed necessary (right)

The application tests also highlighted the necessity of using spacers in order to prevent the squeezing out of the adhesive and to ensure that the bricks stay in position until the adhesive sets (hardens). Accordingly, spacers in the form of transparent or white double-sided tape (series VHB by 3M™) were applied at the edges of the glass bricks to further control the desired thickness of the adhesive layer (see Fig. 9). In specific, the applicability tests showed that:

- The ideal thickness of the (double-sided tape) spacers varies per adhesive. Adhesive A requires a thin spacer (1 mm) since it is very fluid and its gap filling capacity is set to 1 mm. Adhesive C is gummy in texture and requires 2–3 mm; whereas B needs 1.5–2 mm.
- Adhesives B (Teroson MS 9399) and C (Dowsil Experimental Fast Curing Adhesive) took a longer time to set compared to A (3M™ DP610); this is in agreement with the data provided by the manufacturers.
- In terms of cleaning overflow, adhesive A is harder to clean; it sets relatively quickly and obtains a sticky texture. Adhesives B and C are very easy to clean with propanol; even after curing, any excessive adhesive can be easily cut or scraped off.
- Adhesive A yields a fully transparent bond (see Fig. 8); adhesives B and C offer a homogeneous, white bonding surface (see Fig. 9).

### Shear tests

**Fig. 10:**
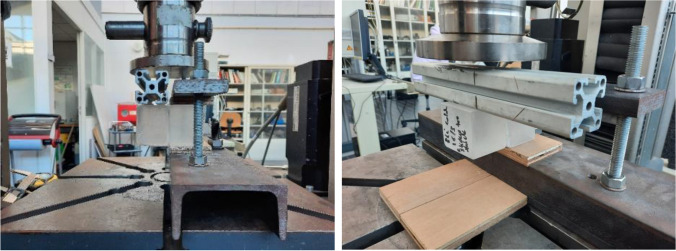
Experimental set-up of the shear tests

In order to further investigate the bonding strength and failure mode of each alternative, the three final adhesive candidates (A, B and C) are tested in shear.^11^ Aim of the tests was to investigate the strength of the bond between the adhesive and the cast glass blocks, as they present considerable imperfections (and thus variations in the adhesive thickness) compared to standard float glass; in addition the chemical response of the adhesive to the cast glass substrate can vary compared to float glass due to alternations in the chemical composition of the glass and traces of the mould material to the glass surface. Two series of shear experiments were conducted under standard laboratory humidity levels, (i) at lab temperature conditions (approx. 20^o^ C) and (ii) at -5^o^ C in order to observe if a drop in temperature influences the strength of the adhesive bond.^12^ Accordingly, triplets of specimens were prepared per test per adhesive candidate, each consisting of two cast soda-lime silica glass bricks of circa 55*55*50 mm bonded together along the entire 55*50 mm surface. The glass samples were cut in-size out of larger glass blocks provided by Wonderglass,^13^ using a water-cooled rotary diamond wheel cutter. The cut surfaces were ground with a 60git diamond plate. The adhesives were applied on the glossy (non-cut) surfaces; double-sided tape spacers were applied at two sides of each sample to control the adhesive thickness.^14^ The specimens for test series (i) were bonded and tested under room temperature after full curing occurred (> 24 h); whereas the specimens for series (ii) were bonded at 7^o^ C; after curing (24 h) they were frozen overnight at -20 °C and then transferred with the use of a portable freezer directly to the testing machine; a temperature sensor was used, indicating that the temperature of the specimens during the test was approx. -5 °C. The tests were performed in a Zwick Z10 displacement controlled universal testing machine under a max. load of 100 kN. A specially manufactured steel frame is used to clamp the glass assembly to the base and restrain any movements. Two 10 mm hardwood plates were placed in between the glass block and steel plates of the experimental set-up to reduce the risk of local peak stresses generation and the subsequent fracture of the glass blocks. The vertical load is introduced by the displacement of the crosshead against an aluminium tube and then on the brick in a speed of 1 mm/min. The experimental set-up can be seen in Fig. 10 below.

### Results

**Fig. 11:**
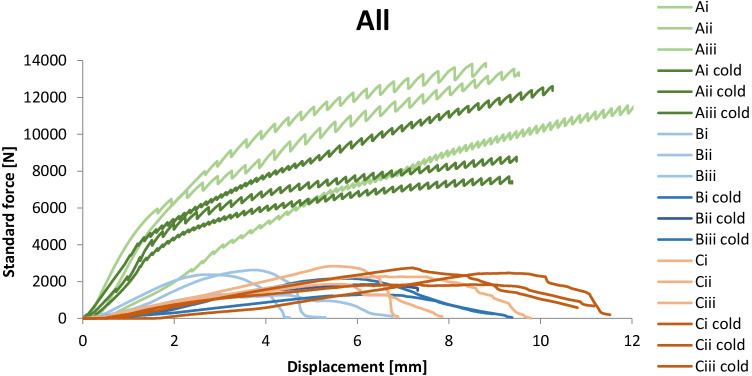
Standard force–displacement graph for shear specimens. The higher rigidity and strength of adhesive A in comparison to adhesives B and C can be observed. The presented displacement refers to the movement of the machine and should not be used to derive the elongation at break of the adhesives or the shear stiffness of the assembly

**Fig. 12:**
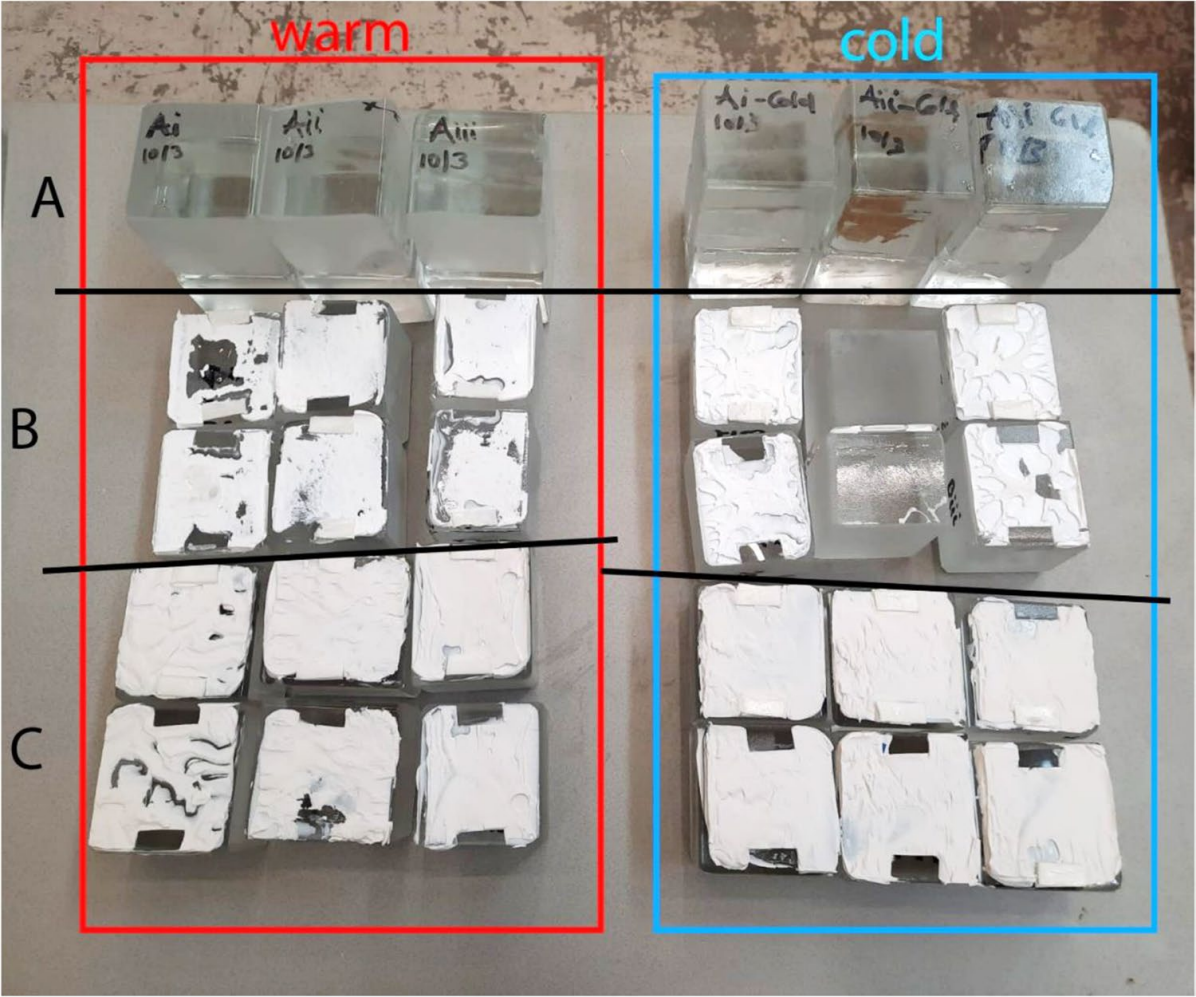
All specimens of the warm (left) series and cold (right) series after being tested in shear

**Fig. 13:**
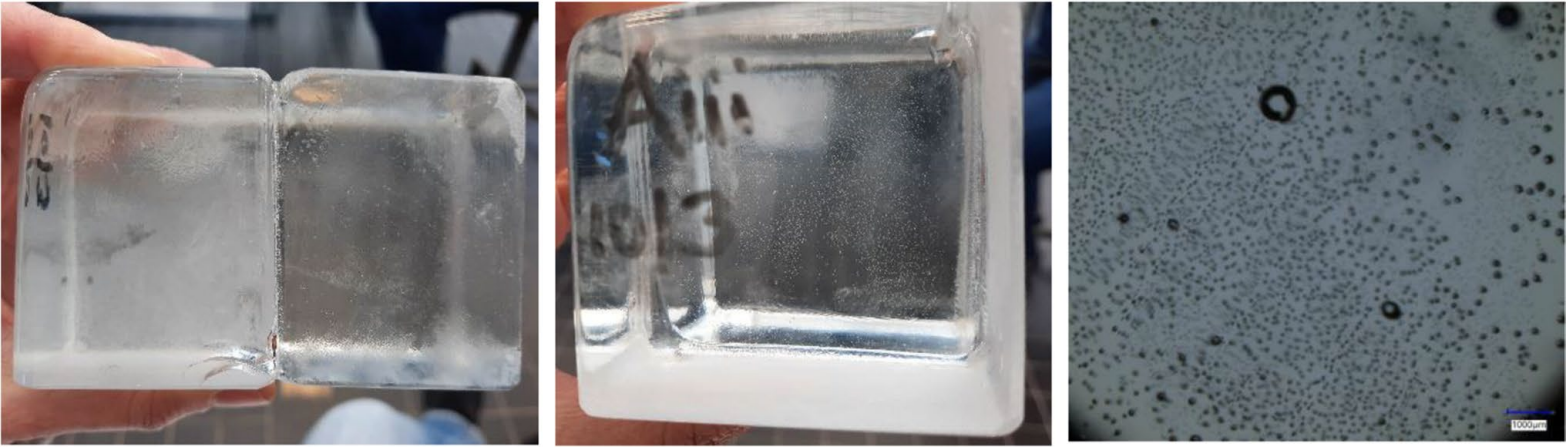
Samples bonded with adhesive A; the tests were interrupted due to bending of the set-up and cracks on the glass bricks (substrate failure, image on the left). The specimens also presented dissipative failure, in the form of miniscule bubbles within the adhesive layer (middle image). Right: miniscule bubbles in the adhesive as observed with a Keyence VHX -7000 digital microscope

**Fig. 14:**
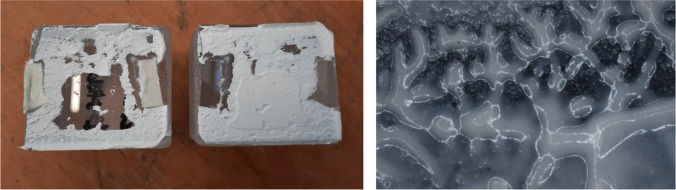
Observed failure mode for adhesive C is mainly cohesive. Right: cohesive damage observed with a Keyence VHX-7000 digital microscope

**Fig. 15:**
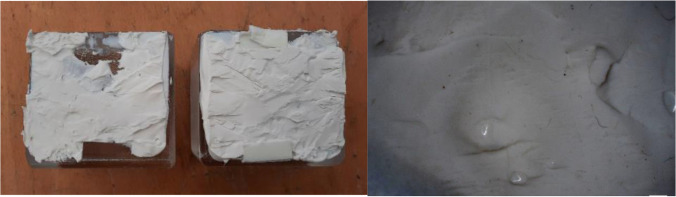
Samples bonded with adhesive B presented either cohesive-adhesive or cohesive failure. The figure on the right shows such a zone of mixed failure, as observed with a Keyence VHX-7000 digital microscope

The standard force vs displacement (F-u) graph of all specimens can be seen in Fig. 11 below. The displacement in Fig. 11 refers to the movement of the displacement-controlled universal testing machine and includes the deformation of the hardwood, as well as errors attributed to the machine itself. Thus, it cannot be used as a reference value for deriving the elongation of the specimens or the stiffness of the assembly. However, the resulting curve can be used as a comparative indication of the overall stiffness per adhesive system and indicate if alternations in stiffness occur with temperature deviations. The summary of the results can be found in Table 4; all tested specimens can be seen in Fig. 12. The following main conclusions on strength and fracture mode can be drawn from these experimental series:

- The following modes of (joint) failure in shear are relevant in our case:
  - adhesive failure, when separation occurs visually at the adhesive/glass brick surface,
  - cohesive failure, when separation occurs visually within the adhesive layer,
  - cohesive-adhesive failure, which is a combination of the previous two modes.
  - substrate failure, when fracture in the glass brick occurs
  - dissipative failure, when microflaws (e.g. bubbles) appear in the adhesive after large absorption of energy.
- The applied forces in all specimens of the B and C series did not result in visible bending of the set-up, thus, it is considered that in these tests, the contribution of bending was minor. In specimens of the A series there was visible bending occurring in the set-up due to the higher applied forces. Therefore, the anticipated engineering shear strength is considerably higher than the one reported in this study.
- Overall, adhesive A presents a considerably higher strength and stiffness compared to the silicate-based B and C. In specific, bond failure of the A specimens did not occur within the given set-up. The experiments had to be stopped due to bending of the experimental set-up, implying as well a higher shear strength than the one reported in this study. Minor cracking (i.e. substrate failure) appeared in the corners of some of the glass bricks; this is attributed to the manual cutting of these bricks in size and lack of fine polishing. In addition, miniscule bubbles (see Fig. 13) were observed in the adhesive layer (dissipative failure) caused by the developed shear stress. These miniscule bubbles are not considered to compromise the strength.
- Adhesives B and C present similar shear strength with adhesive C being marginally stronger. It should be noted that the family of DOW fast-curing silicone adhesives on which adhesive C is based, reach their maximum strength on the 7^th^ day of curing (at 23 °C). A colder temperature during bonding and curing can delay this process. The tensile strength of adhesive C after 7 days has been estimated to 2.05 MPa by DOW Silicones Belgium [13]. Hence, it is anticipated that adhesive C probably exhibits a relatively higher strength than the one derived from the tests if the specimens were tested many days later.
- Adhesive C mostly exhibited cohesive failure (see Fig. 14), while the B specimens typically presented cohesive-adhesive failure (see Fig. 15).
- A marginal drop of strength is observed at the cold series for adhesive B (see Table 4); nonetheless, the number of tested samples is not sufficient for conclusive answers.
- The shear strength of adhesive C appears to be stable within the temperature range of the warm and cold test series.
- Regarding adhesive A, results are inconclusive regarding the effect of temperature as the tests were stopped before failure. The higher strength and stiffness of this adhesive is, however, evident from the force–displacement graph (Fig. 11). Previous tensile tests on shoulder bars by the Institute of Building Construction, Technische Universität Dresden^15^ further suggest that the tensile strength of adhesive A is considerably higher at -20˚C (~ 50 MPa) than at room temperature (~ 10 MPa); tensile tests by Weller and Wünsch [20] on point fixings made of glass and stainless steel before and after various types of artificial aging show good aging resistance of the bonds with DP 610. The 5% fractile values of the residual strength are (except for aging with surfactants) between 2 and 12 MPa.

**Table 4:** Summary of shear tests

Adhesive	Specimen	Type of failure	Joint thickness	bonded area	F_max_	Failure stress	Failure stress* τ_max_
			mm	[mm^2^]	[N]	[MPa]	[MPa]
3M™ DP610	Ai	test interrupted due to the bending of the experimental set-up/cracks observed in glass bricks**	1	2968	13,856.7	> 4.67	> 4.63
	Aii		1	2808	13,544.0	> 4.82	
	Aiii		1	2646	11,648.4	> 4.40	
	Ai cold	test interrupted due to the bending of the experimental set-up/cracks observed in glass bricks**	1	2700	7700.4	> 2.85	> 3.73
	Aii cold		1	2646	12,606.0	> 4.76	
	Aiii cold		1	2448	8781.5	> 3.59	
Teroson 9399	Bi	cohesive-adhesive	2	2192	2629.3	1.20	1.02
	Bii	mainly cohesive	2	2072	2385.3	1.15	
	Biii	cohesive-adhesive	2	1864	1300.3	0.70	
	Bi cold	cohesive	2	2328	2170.0	0.93	0.78
	Bii cold	cracks in glass brick/test stopped	2	2144	1847.1	(0.86) N/A	
	Biii cold	Cohesive-adhesive	2	2348	1297.8	0.55	
DOWSIL Experimental Fast Curing Adhesive	Ci	cohesive-adhesive	3	2469	1869.2	0.76	0.99
	Cii	mainly cohesive	3	2474	2286.4	0.92	
	Ciii	cohesive	3	2181	2843.0	1.30	
	Ci cold	cohesive	3	2392	1843.9	0.77	0.99
	Cii cold	cohesive	3	2516	2746.7	1.09	
	Ciii cold	cohesive	3	2258	2467.1	1.09	

*estimated as nominal value of failure load/effective bonded area

**Specimens in the A category presented minor dissipative damage in the form of miniscule bubbles, and occasional substrate damage in the form of glass corner chipping. The chipping can be prevented with sufficient annealing and fine polishing of the glass bricks

### Final adhesive selection

Taking into account the structural performance of the adhesives and their gap filling properties, it was determined to use both adhesives A and C in the construction of the pavilion as follows:

Based on the results, it could easily be suggested that adhesive A, which presents the highest strength and stiffness and is the only candidate that is transparent in colour, should be applied in the entire construction. Nonetheless, in practice, it was determined to apply the A adhesive only at the bottom rows of the construction where a higher strength would be the most beneficial, as the overlap of the bricks, and thus the bonding surface, was smaller. We opted out of applying this adhesive in the entire construction due to the following practical limitations: (1) this adhesive has a limited gap filling capacity of 1 mm; thus, it could accommodate manufacturing tolerances in the first rows of the pavilion, but as the construction ascends, the manufacturing tolerances could result in a sizeable offset in the height or width of the entire construction that could not be accommodated within 1 mm joints; (2) in addition, the total amount of this adhesive in stock within Europe prior to the beginning of the construction^16^ was insufficient for the entire pavilion.

Adhesive C, DOWSIL Experimental Fast Curing Adhesive, was chosen for bonding the rest of the construction. Compared to adhesive B, it has a similar visual result, yet it presents a relatively higher strength and cohesive failure. Moreover it has speed-curing properties specifically engineered for this project. Equally importantly, it presents the largest gap filling capacity; it can accommodate manufacturing and construction tolerances up to 3 mm. Such a gap-filling capacity is crucial for absorbing within the adhesive thickness the built-up dimensional tolerances towards the higher (top) layers of the construction. The R&D team of Dow Silicones Belgium has specifically optimized this adhesive for this project in order to provide durable adhesive bonding and sealing for components which exhibit different thermal expansion rates, allow for fast homogeneous cure throughout the adhesive cross-section and for an early adhesion development. In specific, both the colour (to white) and the mixing ratio of the two-component adhesive to 4:1 in order to optimize the speed of reaction, have been explicitly adapted for this project. In standard temperature and humidity conditions, the snap time for this mix occurs after 4—6 min and the tack free time lies between 16—18 min which leads to limited sagging properties.

### Visual prototype.

A small 0.8*0.8 m prototype of the wall construction was realized at the TU Delft Glass lab together with the project’s architect at standard laboratory temperature and humidity conditions. A 10 mm thick stainless steel plate of the appropriate curvature was bolted on steel supports, to form the base of the glass structure.

**Fig. 16:**
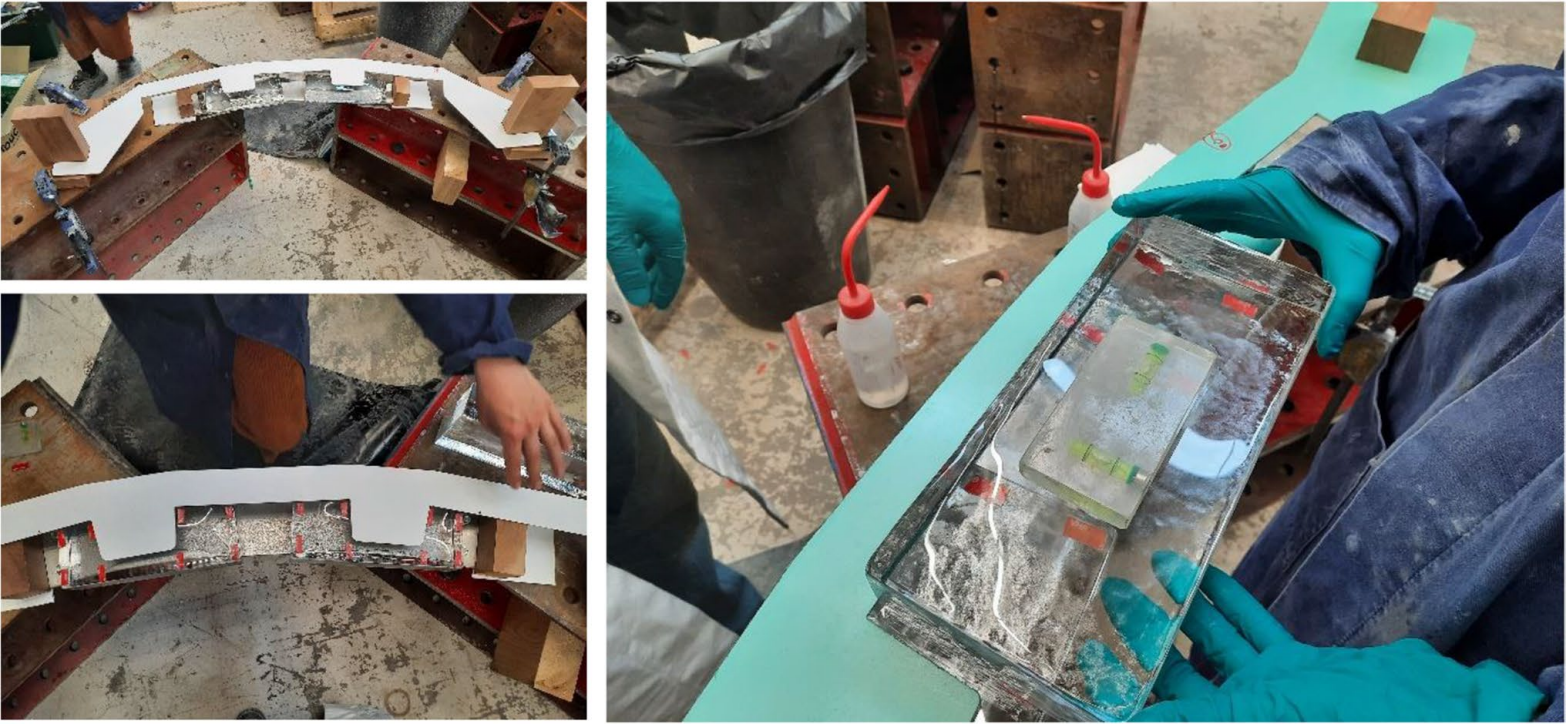
Templates and wooden guides used to indicate the correct position of the glass bricks (left). Levelling of the glass brick with the help of a water-level and additional 0.5 mm double-sided tape stickers (right)

Wooden vertical guides were placed on the sides, on which CNC-cut PVC templates for each row were aligned, to indicate the correct position for the glass bricks. In this manner the extent of the cantilever of each row could be controlled. All bricks had been measured in thickness using a manual calliper, and categorized in groups per 1 mm difference in height (from 51 to 55 mm). Before bonding, the bottom surface was thoroughly cleaned using 2-isopropanol and double sided tape stickers were placed at the corners indicated by the template. The colour of the tape was matched with the colour of the adhesive. While the cover of the double-sided tape was not yet removed, a brick would be placed and adjusted using a spirit level (see Fig. 16). In case of considerable unevenness of the glass brick or built-up deviations, an additional, 0.5 mm thick, double-sided tape was added where required to level the top surface of the brick, or a different brick was used. One height category was used per row to minimize deviations. When all bricks were selected per row, they were removed, and all surfaces to be bonded were cleaned using 2-isopropanol. The cover of the stickers corresponding to the first brick to be bonded were removed, and adhesive was dispersed in an “X” shape within the limits of the stickers. The brick was then rapidly placed in position and secured in place using minimum hand pressure. The same process would be repeated with each brick. To avoid the excessive use of nozzles and restrict this to one nozzle per row, a small amount of adhesive was pumped out every 30 s to ensure that no curing would start to occur within the static mixer of the nozzle.

**Fig. 17:**
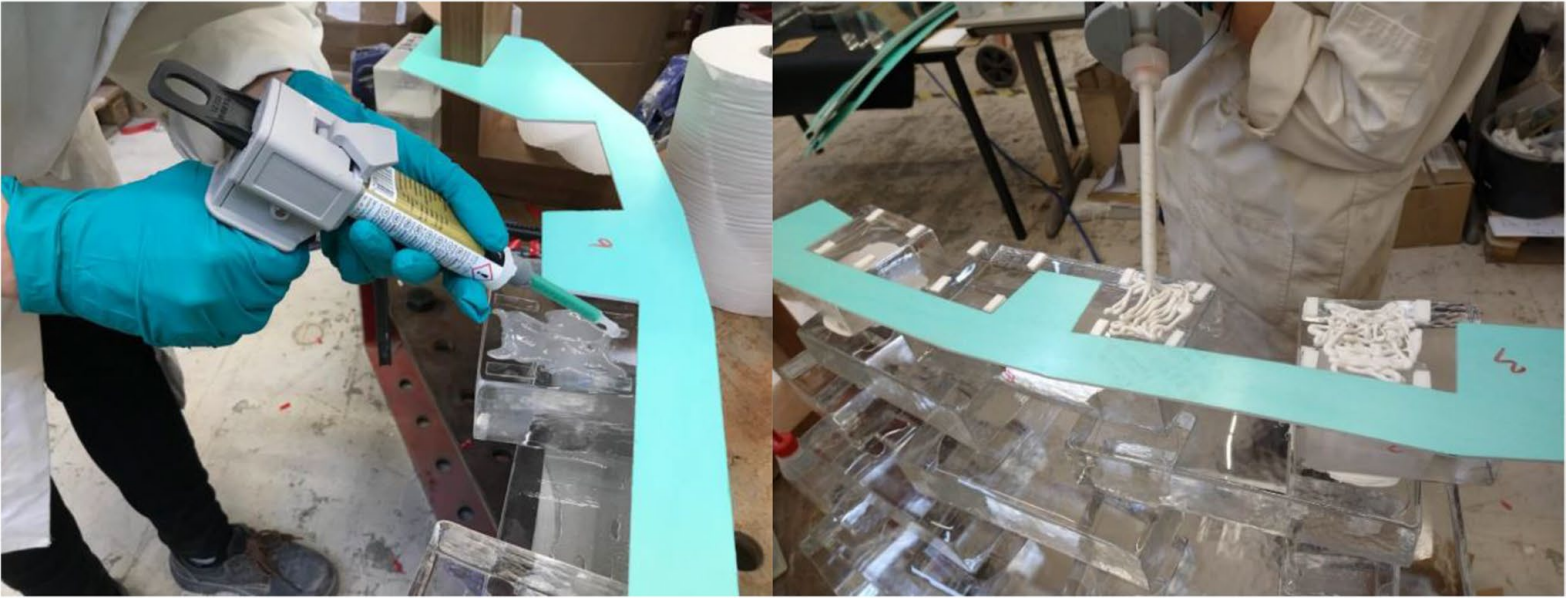
Application of the 3M™ DP610 adhesive (left) and DOWSIL Experimental Fast Curing adhesive (right) during the construction of the prototype. Double-sided tape of clear (left) or white (right) colour were used to fix the bricks in place until the glue had hardened and to control the thickness of the adhesive. CNC-cut PVC templates (one design per row) were used to indicate the correct positioning of the glass bricks

The prototype showed that the application of both adhesives was simple and fast (Fig. 17). In both cases, the dispersing and brick placement had to be done within a time frame of 3–5 min, to ensure proper bonding. Thanks to the double-sided tape spacers, the bricks would be instantly fixed in position. A 1 h interval prior to continuing to the next row was required in the case of the 3M™ DP610, while a 2 h interval was found sufficient for the DOW adhesive. The cleaning of adhesive overflow, although relatively easy with the use of 2-isopropanol, proved to be time-consuming. Therefore, to simplify the construction process, the dispersion of the adhesive in a round blob shape was opted in the final structure (instead of the X-shape application in the prototype). The mock-up also further confirmed that the joints bonded with the white Fast-Curing Experimental Adhesive by Dow (Adhesive C) may be visible from top view but cannot be easily seen from the side view; thus the white colour is not considered visually obstructive.

**Fig. 18:**
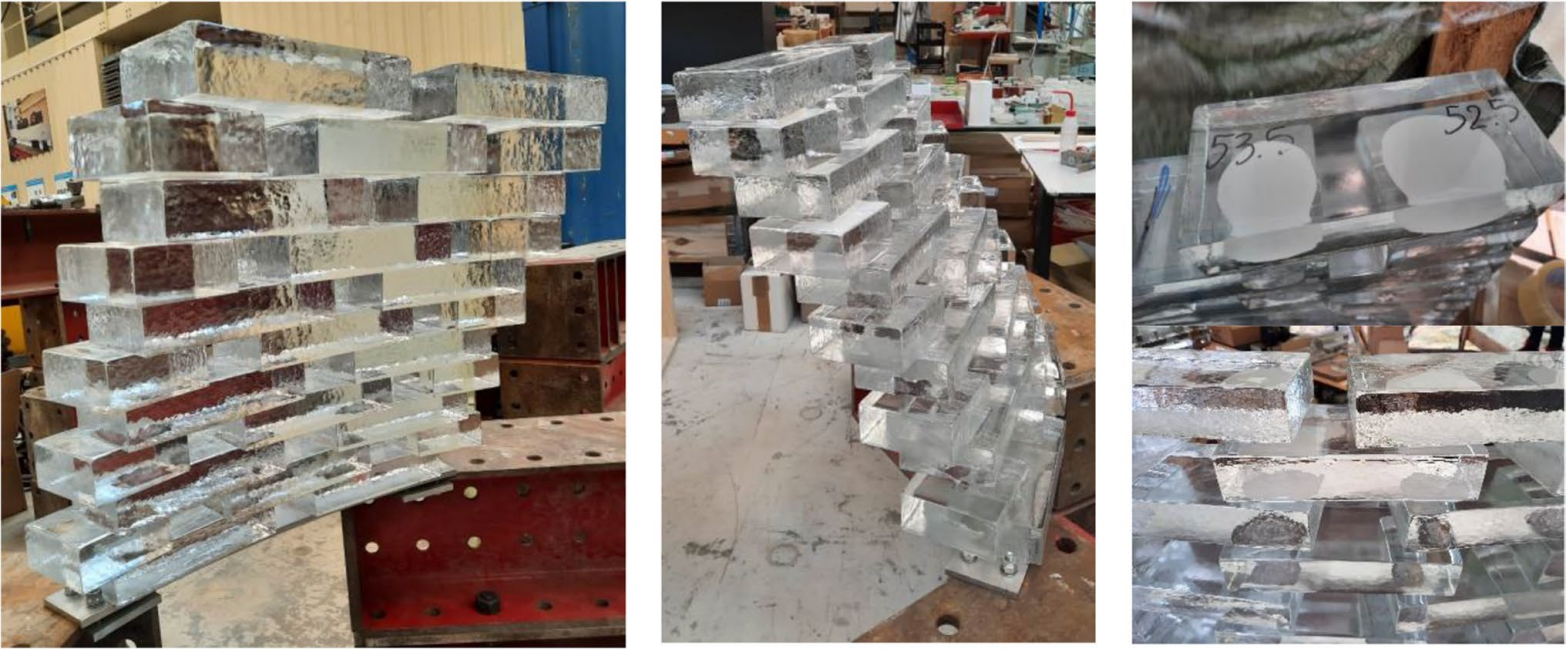
Prototype constructed at the TU Delft Glasslab, showing the natural tilt of the bricks due to the larger cantilevering width in the initial design (left and center). The application of the DOWSIL Experimental Fast Curing Adhesive in blobs may be visible from top view (top right), but cannot be easily seen from the side view (bottom right), and is thus not obstructive

More importantly, the mock-up provided evidence that the initially designed cantilevering gap between each row, set at + 25 mm, would be challenging as it was leading to a natural tilt of the bricks in the structure (Fig. 18). Consequently, it was decided to limit the cantilever gap per row at a maximum of 10 mm to prevent this action. No bending was observed at the steel base or delamination at the glass bricks of the first row.

## Construction

### Construction site set-up

**Fig. 19:**
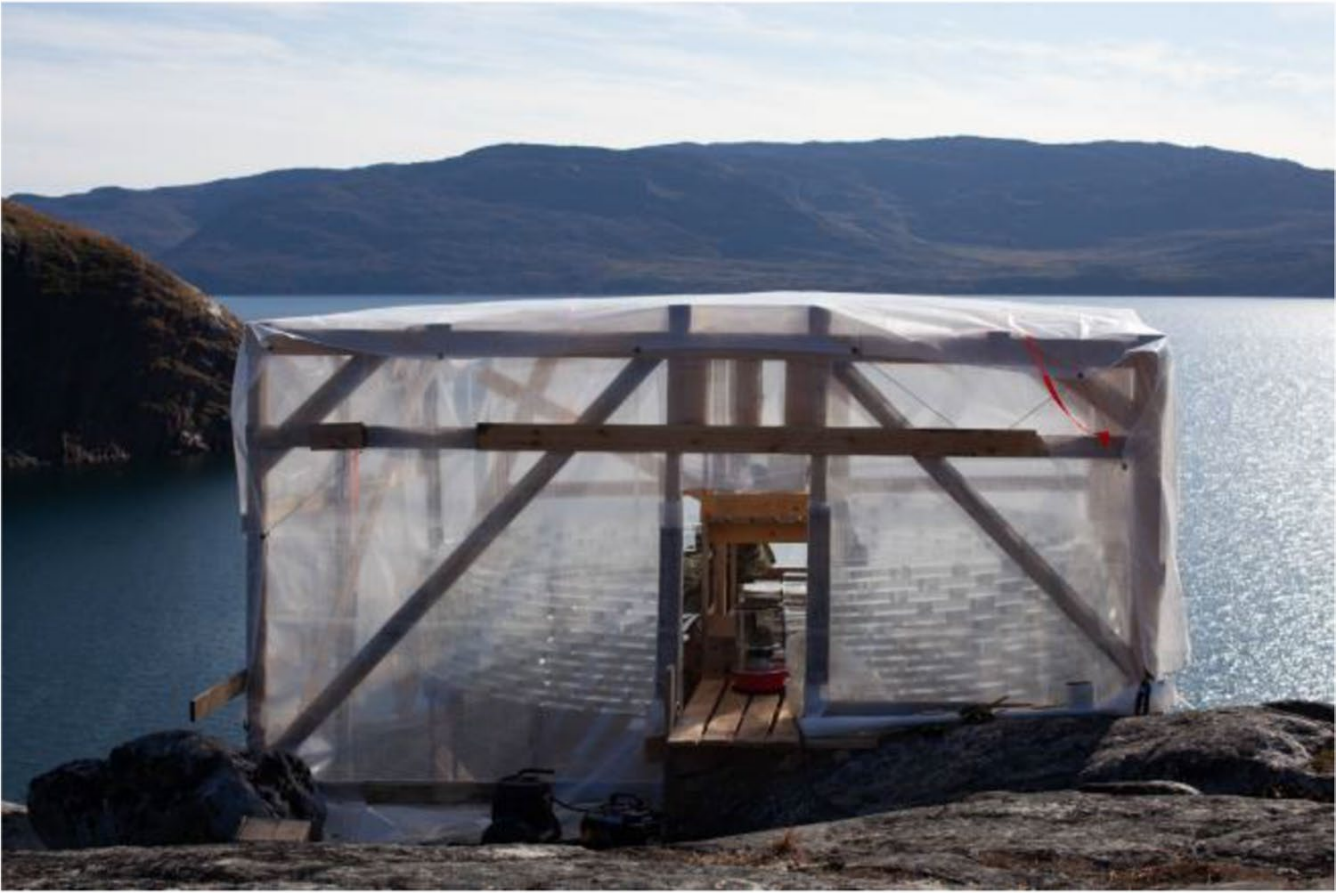
The tarpaulin tent covering the construction. Photo credits: K. Ikonomidis

**Fig. 20:**
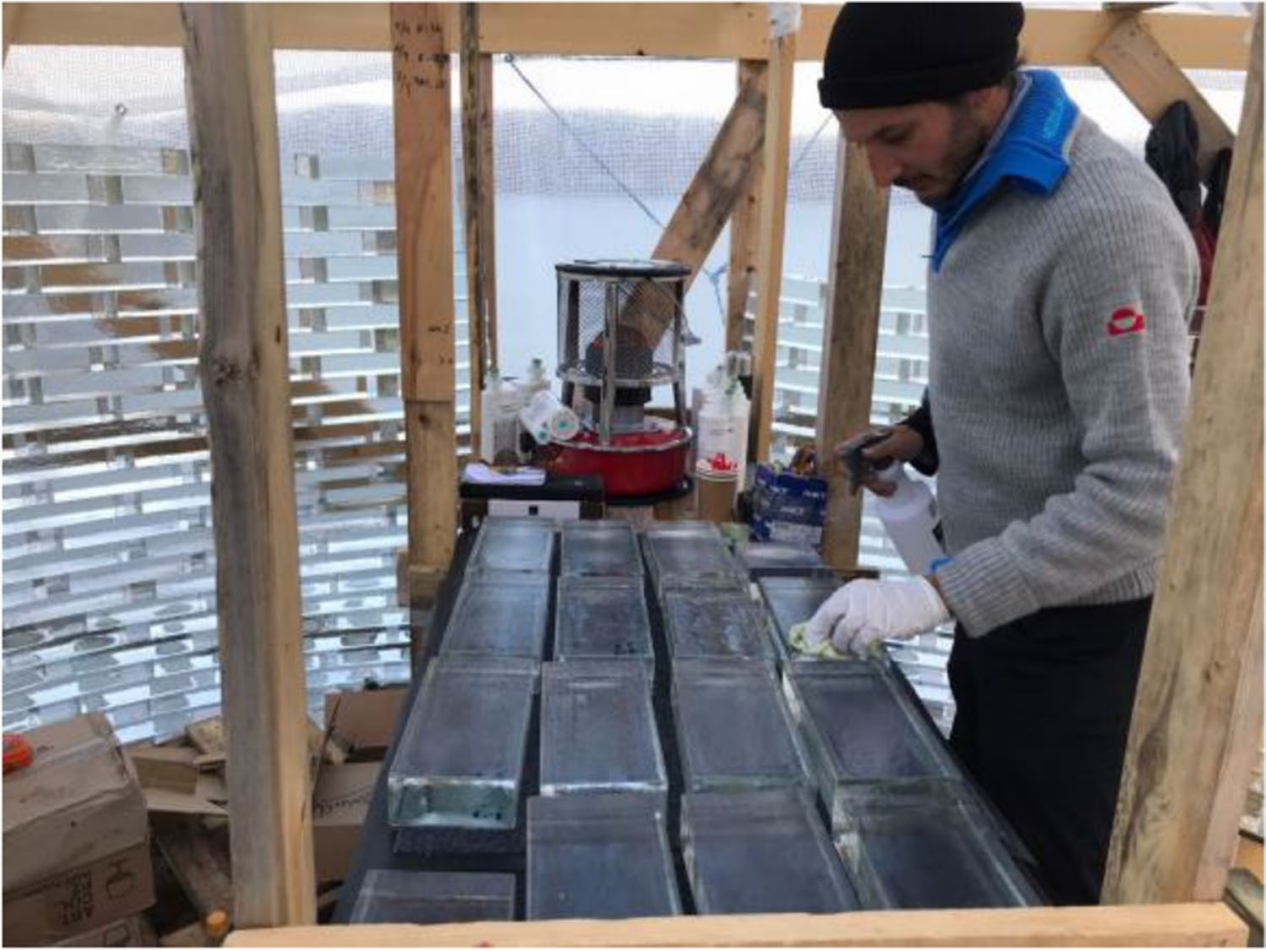
The working platform (and gas-stove) installed in the middle of the construction-site. Photo credits: K. Ikonomidis

**Fig. 21:**
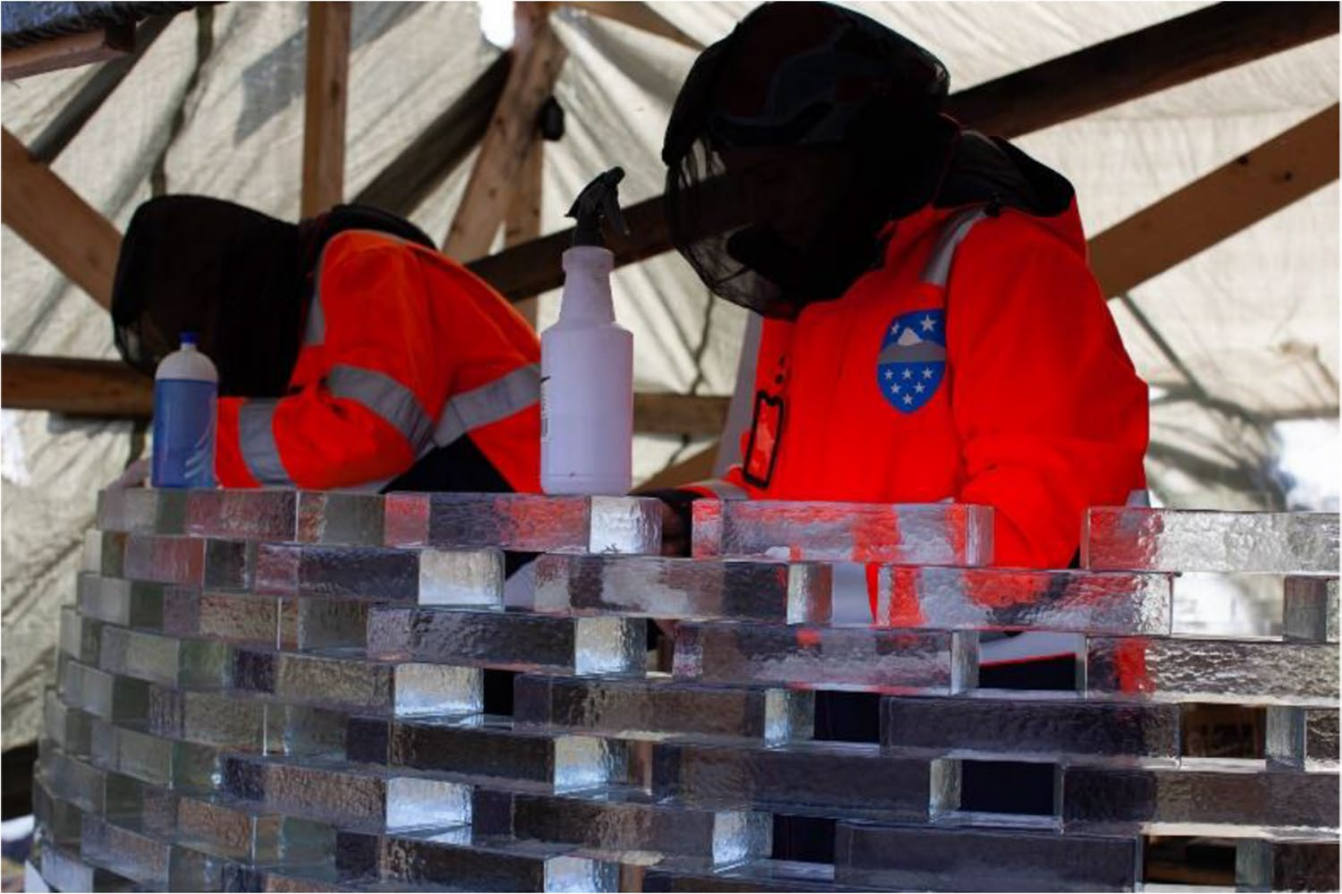
The two TU Delft researchers bonding glass bricks on-site. Besides the adverse climate conditions, mosquitos proved to be an equal challenge in Greenland, deeming the use of mosquito nets necessary

All material was initially shipped in the town of Sisimiut, from where it arrived via charted boat to the harbour of Sarfannguit. ATVs (quad bikes) were used to transport the material to the pavilion’s site, which is approx. 1 km uphill from the settlement’s harbour. A tent using locally available water-resistant, tarpaulin plastic fabric was erected to protect the construction from adverse weather conditions and dust (Fig. 19). In the middle of the site, between the two walls, a wooden working platform was installed, where the bricks of max. two full rows of construction were loaded (see Fig. 20). A portable gas stove was also installed for additional heating. The adhesives were stored in the local guesthouse so as to remain in ambient temperature. Each day, the necessary amount for bonding was transferred to the construction site. An additional tepee tent was installed in close proximity to the pavilion’s site in order to store overnight all other tools and equipment needed, such as clamps, drills, adhesive dispensers, etc. The glass blocks were placed in three wooden pallets covered by tarpaulin fabric next to the pavilion’s site (see Fig. 4) and were gradually transported to the site upon demand. The first 12 rows of the construction were built by a team of four: the architect, two TU Delft researchers and a local resident of the village (see Fig. 21). After the departure of the TU Delft researchers, the construction continued by the architect together with two residents of the settlement. The bonding of the glass pavilion started on 8^th^ of August 2021; the pavilion opened to the public on the 3^rd^ of October 2021.

### Bonding

**Fig. 22:**
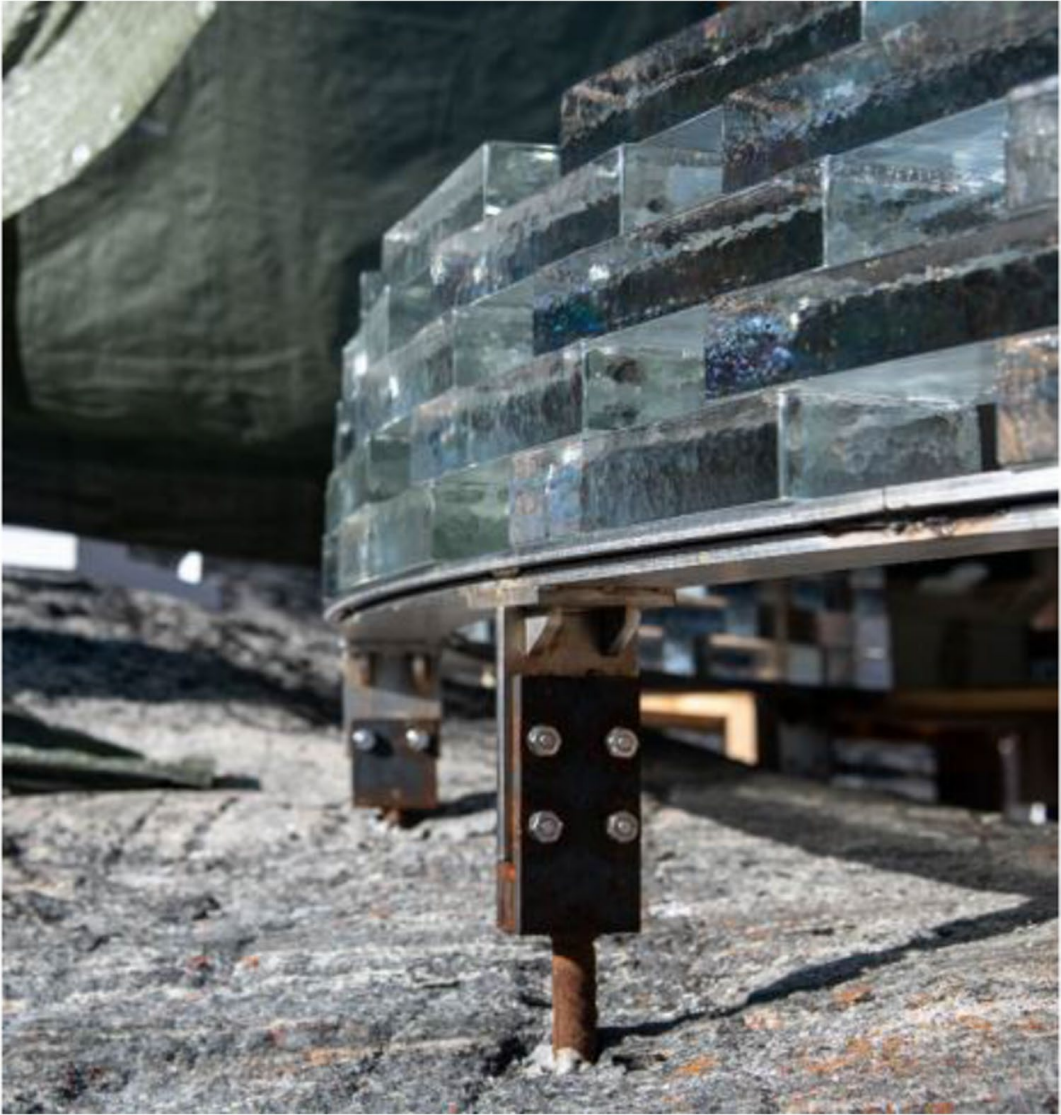
Foundation of the glass-brick Qaammat Pavillion

The erection of the glass structure started on top of two welded 10 mm thick, arc shaped steel plates that were levelled. The plates are directly supported by stainless steel bars bonded in drilled holes on the rock below (see Fig. 22); a method borrowed from local house building traditions. Each row per wall comprises 14–16 bricks. The bonding of the glass brick structure occurred during the months of August and September with mean outdoor day temperatures 11˚C and 3˚C respectively and an average air humidity of 70%.

In specific, the entire application of the 3 M™ Scotch-Weld™ Polyurethane Adhesive DP610, used for the bonding of the first 9 rows, was done from the 8^th^ until the 15^th^ of August, when air temperature during the day remained between 12–18˚C. The application of the DOWSIL Experimental Fast Curing adhesive was done from the 15^th^ of August onwards; even though temperatures dropped in September, the silicone-modified adhesive presents rather stable properties; the cartridge had to be stored in relatively warm temperature^17^ prior to its application so that the adhesive can be easier dispensed and the tent was acclimatized with the gas stove. Overall, the actual construction time was less than 6 weeks; during days with strong winds and rain the construction would stop.

**Fig. 23:**
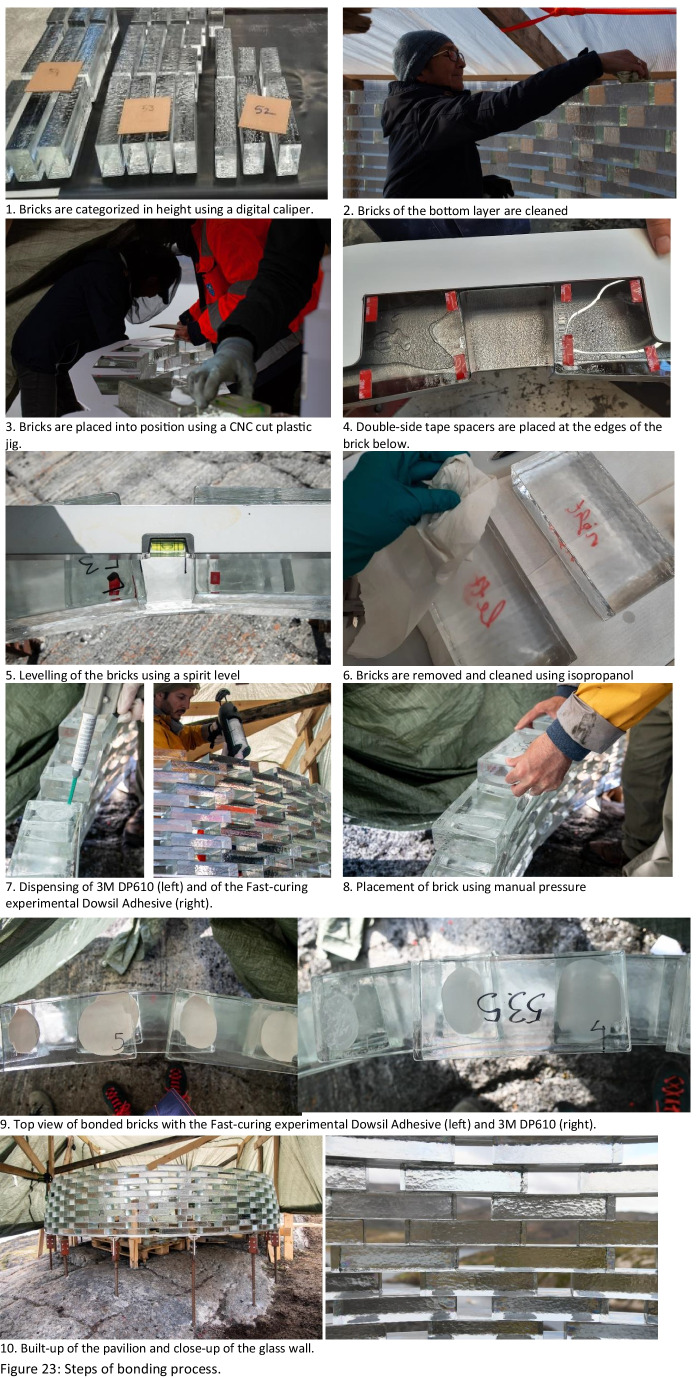
Steps of bonding process

As previously mentioned, the first 9 rows of the construction are bonded using the 3M™ Scotch-Weld™ Polyurethane Adhesive DP610 (adhesive A), whereas the remaining structure was bonded by the DOWSIL Experimental Fast Curing Adhesive by Dow (adhesive C). The first row of glass blocks is directly bonded by 3M™ Scotch-Weld™ Polyurethane Adhesive DP610 to the stainless steel plates. To prevent the development of considerable fluctuations in the height of the construction, which could not be accommodated within the chosen adhesives’ thickness, a similar yet simplified approach to the one followed at the construction of the Crystal Houses façade [2] was followed. The steps of the bonding process are shown in Fig. 23:

1. Initially, all bricks are measured using a digital calliper at the two edges along their length and sorted according to height deviations of 0.5 mm. Bricks with height differences up to 1 mm are used in each row.
2. The top surface of the brick row below is first cleaned with 2-propanol.
3. A special CNC-cut plastic jig is used to mark the position of the top bricks.
4. Strips of transparent double-sided 3M™ tape of (a) 1 mm thickness for the first 9 rows, where 3 M™ Scotch-Weld™ Polyurethane Adhesive DP610 is applied and of (b) 2 mm thickness for the remaining structure, where the DOWSIL Experimental Fast Curing Adhesive is used, are then applied as spacers at the four corners of the outline of each top brick in order to guarantee the joint dimension before cure of the adhesive and avoid any squeeze out due to the glass brick weight. The tape is bonded to the bottom glass brick row. The protective cover of the top surface of the tape is not yet removed.
5. Prior to bonding, all glass bricks of a new row are laid down – bricks of similar height as described in step 1 are used in each row. A spirit level is used to check their levelling of each two adjacent top bricks. If the bubble at the spirit level is not in the centre, the corresponding brick is replaced with another one that accomplishes better levelling in the specific location. The final selection of bricks is then numbered to guarantee their correct bonding sequence.
6. The marked bricks are removed and cleaned by 2-propanol. The top protective cover of the spacer tape is now removed.
7. The adhesive is dispensed with the aid of a manual gun in the shape of two circular blobs on the surface of the respective two adjacent bricks of the bottom layer.
8. The top (marked) bricks are then placed using manual pressure for approx. 10 s, so as to ensure that the adhesive spreads evenly. During the curing of the adhesive the top bricks are held in position as they are attached to the double-sided tape spacers.
9. Upon completion of one row, any excessive adhesive (overflow) is cleaned with the aid of 2-propanol.

Adhesive A, 3M™ DP610, comes into cartridges of 48.5 ml and can be easily applied by a small manual dispenser (see Fig. 23, step 7). Two of the crew members were applying the adhesive using two adhesive dispensers, whereas the other two would place the glass bricks and clean any excessive overflow.

In the case of Adhesive C, DOWSIL Experimental Fast Curing Adhesive, the adhesive is packed into cartridges of 400 ml in 1:4 mixing ratio and is quite thixotropic, rendering its extrusion via a manual dispenser through an 18-element, graduated mixing nozzle, quite force-intensive. DOW Silicones Belgium had recommended the use of a pneumatic dispenser; nonetheless, this was not possible at the construction site due to the lack of electricity or of a generator. A good alternative was to order a battery-driven dispenser; however, after contacting multiple providers in EU and USA, we could not secure a battery-driven dispenser for such cartridge and mixing ratio in stock before September. Thus, given the limited timeframe for the building of the pavilion and the lack of alternatives, the construction started with the application of the adhesive via a manual dispenser for the first few rows (3–4); in September, a battery-driven dispenser was obtained and the application of the adhesive was much easier and faster. In any case, due to the prolonged time needed for the DOWSIL Experimental Fast Curing adhesive to obtain its full strength, a maximum of 3 rows per wall was bonded each day.

**Fig. 24:**
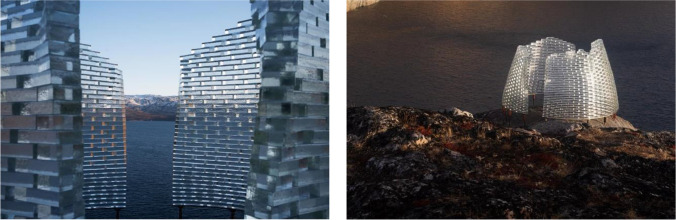
The completed pavilion. Photo credits: Julien Lanoo

**Fig. 25:**
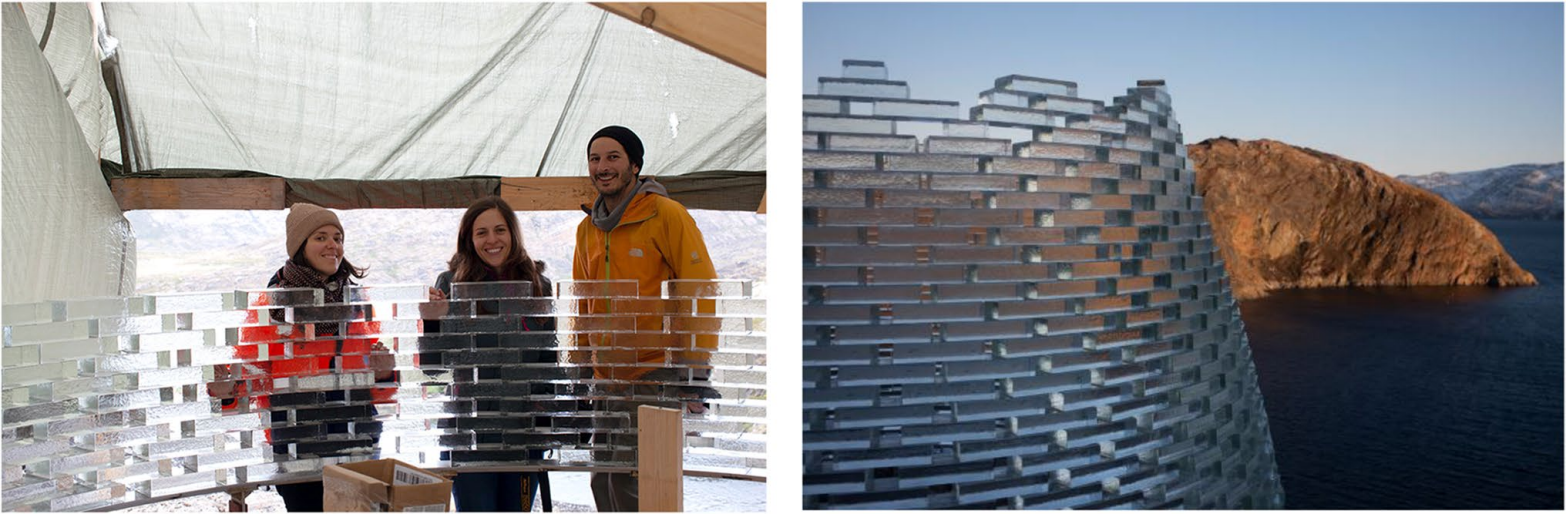
Left: The TU Delft researchers together with architect K. Ikonomidis, building on-site. Right: Close-up of the completed Qaammat Pavilion

Each day, once the bonding process was completed, the two glass walls were wrapped tightly using a plastic fabric to prevent the contamination/embedding of the joints with dirt, humidity, etc. The sealing of the construction took place after the glass walls were completed by applying via a battery gun Sikaflex 112 Crystal Clear, a clear sealant. This sealing is essential in order to avoid water getting into the joints and freezing which can eventually lead to breakage of the glass brick structure. The completed pavilion can be seen in Figs. 24 and 25.

## Conclusions

Overall, the realization of the Qaammat pavilion showcases a new direction of constructing with cast glass components. Built in a location characterized by remoteness and extreme weather conditions and realized with limited budget and resources, the Qaammat Pavilion exemplifies the versatility of cast glass as a building material and its great architectural potential. Its construction further demonstrates that besides strength and visual properties, ease-of-assembly is equally important for adhesively bonded cast glass assemblies. Here, the key challenge was ensuring the desired aesthetic appearance and structural integrity of the glass structure, whilst solving the technical and installation complexity of a bonding solution, which would fulfil the performance and durability requirements linked to the artic climate and allow for a simplified and fast assembly process, suitable for non-professional builders.

This bonding approach is quite the opposite to the assembly process used so far in relevant examples. Hence, the pavilion’s construction showcases that the choice of adhesive family is highly dependent on the prerequisites set for each case-study. Acrylates and epoxies are preferred for applications where high-strength and high transparency are crucial (e.g. Crystal Houses façade and Atocha Memorial); yet they call for a high-precision construction and a highly-specialized crew [15]. Here, ease-of-assembly and a demanding range of operating temperatures proved to be the most critical aspects, rendering adhesives from the silicone and polyurethane families as the most suitable candidates due to their increased gap-filling capacity; although their strength is considerably lower yet still sufficient for self-supporting structures.

Mono-component adhesives of these adhesive families were discarded at an early stage as an option due to their temperature- and humidity- dependant curing mechanism. The focus was placed instead on available, two-component adhesives, which perform well under a wide range of temperatures and have a satisfactory gap filling capacity. Applicability and shear tests on a pre-selection of adhesives led to final selection of two adhesives for the construction of the pavilion: 3M™ Scotch-Weld™ Polyurethane Adhesive DP610 which has a shear strength above 4.5 MPa, 1 mm gap filling capacity and is clear in colour; and DOWSIL Experimental Fast Curing Adhesive customized by Dow Silicones Belgium for this project, with a shear strength of circa 1 MPa, 3 mm gap filling capacity and white colour.

The build-up of a visual mock-up with bricks bonded by these two adhesives suggested that the inclination of the walls should be reduced, in order to ensure structural stability and further confirmed the necessity of using double-sided tape spacers to guarantee the desired adhesive thickness and secure the bricks in position until the adhesive sets. The visual prototype also revealed that the application of the white in colour, DOWSIL Experimental Fast Curing Adhesive may be visible from top view, but cannot be easily perceived from the side view.

Accordingly, 3M™ DP610 was selected for the bottom rows of the pavilion where higher strength was required due to the reduced overlapping of the bricks (and thus smaller bonding surface). Tolerances in the first rows of the pavilion are minimal and could be absorbed within the adhesive’s limited gap filling capacity. The DOWSIL Experimental Fast Curing Adhesive was selected for bonding the rest of the construction; compared to 3M™ DP610 it has a lower, yet satisfactory strength, but owing to its considerably larger gap filling capacity it facilitates the ease of assembly. Despite this, the bricks had still to be measured and categorized based on their total height in order to avoid the built-up of construction tolerances larger than the 3 mm gap filling capacity of the DOWSIL Experimental Fast Curing Adhesive.

The build-up of the pavilion in Greenland further highlighted the practical challenges linked to the assembly of an adhesively-bonded glass brick structure in a location characterized by remoteness and extreme winter conditions. Despite the installation of a tent, the lack of electricity and other commodities common in construction sites made it challenging to regulate the temperature and humidity levels within. This in turn, indicated that the entire construction had to be built within a tight time schedule, raising concerns from a logistical point of view. Even though the desired adhesive quantities were secured on-time, obtaining the needed application equipment, such as cartridges, nozzles and battery-driven dispensers prior the start of the construction proved incredibly challenging. Shipping difficulties and limitations, and equipment/electronics shortage due to the Covid-19 pandemic were added complexities that make the development and construction of this project unique.

## Recommendations

Overall, the construction of the Qaammat Pavillion further exemplifies the importance of the adhesive selection at an early design stage in order to realize a structure as close as possible to the envisioned architectural design. Equally importantly the adhesive selection can further prevent or add to the logistical challenges of a design, causing or averting manufacturing delays and construction complications interwoven to the properties of the bonding media. It also reveals that logistical challenges do not only evolve around ordering the main materials on time, such as glass blocks and adhesive; the availability of supporting technical equipment, such as the battery-driven dispenser in our case, is equally critical for the successful construction of the bonded glass structure. In addition, it shows that a close-collaboration between the architects/designers and researchers is crucial for the success of novel cast glass concepts.

The research and development of the adhesively-bonded system for the Qaammat pavilion further confirms the need of experimental validation of structural systems made of adhesively-bonded cast glass components, in order to derive the desired engineering data and ensure their safe structural application, as there is a lack of respective guidelines, building regulations and standardized data [2]. The construction of visual mock-ups is equally important for optimizing the bonding process and resulting visual appearance.

Addressing all the above, a breakthrough adhesive solution for cast glass applications would be the development of a structural transparent foil that allows for in-situ lamination of the components by controllable heating; such a bonding technology would facilitate the construction, equalize stresses in the construction, and would be able to accommodate dimensional deviations and guarantee the desired uniform visual result as well.

## Contribution by the Authors

The design and construction of the pavilion has been assigned to Konstantin Ikonomidis, founder of Konstantin Arkitekter, by Qeqqata Kommunia; UNESCO Aasivissuit – Nipisat. The TU Delft researchers (Faidra Oikonomopoulou, Telesilla Bristogianni and Mariska van der Velden) have generously volunteered the research, development and experimental validation of the adhesively-bonded glass brick system. Faidra Oikonomopoulou and Telesilla Bristogianni also assisted in the construction of the pavilion on-site, co-ordinated and led by architect Konstantin Ikonomidis.

## Footnotes

1 According to [1] the lowest temperature recorded at Sisimiut, a town in close proximity to Sarfannguit, is -35˚C.

2 Lamination was not an option in these examples due to the numerous layers that needed to be bonded.

3 Lower temperatures than the stated margin have been recorded in Greenland (as low as -69.6 ˚C); nonetheless these occur in considerably norther locations than the one of the pavilion.

4 Given the limited height (2 m) of the sculptural pavilion, accumulated construction tolerances were anticipated to be able to be absorbed within a 3 mm thick adhesive layer.

5 As previously discussed, in the Atocha Memorial an acrylate of 2.5 mm gap-filling capacity was custom-developed, but such a solution was not viable in this case given the limited budget and time-frame.

6 the experimental work by [17] also suggested that glass bricks with a sandblasted surface presented a considerably higher shear strength; however, the post-processing of the bricks had to be avoided in our case to keep the budget low.

7 For example, according to [13] DOWSIL™ 993, used for the bonding of the Qwalala structure, develops a sufficient level of cure within 3 days; this reaction time will further increase when the ambient temperature decreases: thus, in Greenland, cure may require as long as several weeks at the expected application temperature of 5–15˚ C, rendering it unsuitable for this specific application. Although this adhesive was initially considered, it was quickly discarded due to the prolonged curing time.

8 Adhesive A requires mixing nozzles, type Teroson ET 6700 statmix small (for 50 ml cartridges); adhesive B requires 3M EPX Square Green Mixing Nozzles, suitable for 48 ml—50 ml volume and 1: 1 or 1: 2 ratio.

9 In general, ensuring consistent mixing is more complex for white silicone as conventional testing such as butterfly or glass tests do not show heterogeneous colours. When using automated dispensing equipment, it is possible to ensure the quantity of base and catalyst is correct through weight.

10 Adhesive (C) requires the use of mixing nozzles with a 10 mm inner diameter and 18 section element (MFHX 10-18 T) and a wide nozzle opening (e.g. ø3mm) in order to prevent excessive pressure building up at the bottom of the smaller catalyst tube cartridge, which in turn could risk tube breakage and leakage, leading to improper mixing of the two components and insufficient curing of the adhesive. A considerably larger pressure force is required to allow the adhesive to flow through these mixing nozzles compared  to the smaller nozzles utilized in options (A) and (B).

11 The performance of a lap shear test was not possible with the available set-ups for the bulky cast glass components, taking into account their voluminous cross-section and imperfect surface compared to float glass. In the discussed set-up bending can occur, as discussed in the results, thus, the shear stress values derived can be seen as conservative. In actual conditions, the structure would be subject to a combination of shear stresses and bending, due to its protruding geometry and lateral wind forces.

12 Ideally the bonded specimens should be tested at -35˚C but this would require a universal testing machine with an incorporated climate test chamber which was not available at the time of this research in the lab.

13 The bricks were cut to a smaller size in order to allow for a smaller bonding surface as the max. load capacity of the testing equipment would not be sufficient to test the specimens until failure if the full surface of the brick was to be tested.

14 The surface occupied by the double-sided tape has been subtracted from each bonded sample in the estimation of the failure stress.

15 Data provided via personal communication of the authors with Dr. Christiane Kothe and Prof. Dr. Christian Louter of TU Dresden.

16 As previously mentioned, the construction of the pavilion had to be completed before October 2021 in order to ensure an exterior temperature at approx. 5 – 15 ˚C. Communication with 3M™ sellers with several EU countries indicated that there was limited stock of this adhesive prior to September. The shortage is partially linked with the limited shelf life of the adhesive, meaning that bulk orders need to be pre-ordered. We could therefore only secure 130 cartridges by the sellers of 3M™ in Italy and Sweden, which would suffice for bonding approx. 9 rows of the entire glass structure.

17 Prior to its application, the adhesive was stored at the nearby tepee tent in plastic containers together with 1.5 L plastic bottles filled with hot-water and covered with blankets in order to be maintained at approx. 20˚C degrees prior to their application.

## References

[1] Kragh J, Pedersen F, Svendsen S (2005) Weather Test Reference Year of Greenland. In: 7th Symposium on Building Physics in the Nordic Countries, Reykjavik. IBRI

[2] Oikonomopoulou F (2019) Unveiling the third dimension of glass. Solid cast glass components and assemblies for structural applications. PhD, TU Delft

[3] EN 572–2:2012 (2012). In: Glass in building - Basic soda lime silicate glass products - Part 2: Float glass

[4] Oikonomopoulou F, Bristogianni T, Veer FA, Nijsse R (2018) The construction of the Crystal Houses façade: challenges and innovations. Glass Structures & Engineering 3:87–108. 10.1007/s40940-017-0039-4

[5] Paech C, Göppert K (2018) Qwalala – Monumentale Skulptur aus verklebten Glasblöcken. ce/papers 2(1):1–12

[6] Hautekeer J (2018) Dow Group: Keeping it Tight. In: Intelligent Glass Solutions, vol. Spring 2018. pp. 34–39. IPL

[7] Bos F, van der Heijden T, Schreurs P (2012) The Glass Sphinx: A Massive Stacked Glass Sculpture. In: Bos F, Louter C, Nijsse R, Veer FA (eds.) Challenging Glass 3. Conference on Architectural and Structural Applications of Glass, Delft, pp. 47–56. IOS Press

[8] Nijsse R (2012) Glass Walls Carrying the Roof and Withstanding the Wind Load on the Facade: Conservatory of the Museum in Dordrecht and Raaks Glass Cube in Haarlem. In: Bos F, Louter C, Nijsse R, Veer FA (eds.) Challenging Glass 3. Conference on Architectural and Structural Applications of Glass, Delft pp. 111–120. IOS Press

[9] Bos F, Van der Heijden T, Geurts R (2014) The glass sphinx : theory to reality. In: Louter C, Bos F, Belis J, Lebet J (eds.) Challenging Glass 4 and COST Action TU0905 Final Conference, Lausanne pp. 551–558. CRC Press Taylor & Francis Group

[10] Paech C, Göppert K (2008) Innovative Glass Joints - The 11 March Memorial in Madrid. In: Louter C, Bos F, Veer F (eds.) Challenging Glass: Conference on Architectural and Structural Applications of Glass, Delft, The Netherlands, pp. 111–118. IOS Press

[11] Oikonomopoulou F, Veer FA, Nijsse R, Baardolf K (2015). A completely transparent, adhesively bonded soda-lime glass block masonry system. Journal of Facade Design and Engineering.

[12] Wurm J (2007). Glass Structures: Design and Construction of Self-supporting Skins.

[13] Hayez V, Aksoy B, Bristogianni T, Oikonomopoulou F, Ikonomidis K (2021) The Qaammat Pavilion. Innovation, collaboration and inclusion. In: Intelligent Glass Solutions, vol. winter 2021. pp. 78–89

[14] Dow Chemical Company (2018) Sustainable Building with Renewable Energy Withstands Extreme Weather. Case Study: Princess Elisabeth Research Station. In. Dow Chemical Company

[15] Oikonomopoulou F, Bristogianni T, Barou L, Veer FA, Nijsse R (2018) The potential of cast glass in structural applications. Lessons learned from large-scale castings and state-of-the art load-bearing cast glass in architecture. Journal of Building Engineering 20:213–234

[16] Barou L, Oikonomopoulou F, Bristogianni T, Veer FA, Nijsse R (2020) Fill-in-glass Restoration: Exploring issues of compatibility for the case of Schaesberg Castle. In: Roca, P., Pelà, L., Molins, C. (eds.) 12th International Conference on Structural Analysis of Historical Constructions SAHC 2020, Spain, pp. 1571–1582. International Centre for Numerical Methods in Engineering, CIMNE

[17] Fíla J, Eliášová M, Sokol Z (2019). Experimental investigation of mortar mechanical properties for glass brick masonry. Glass Structures & Engineering.

[18] 3M Scotch-Weld EPX Adhesive DP610 (1998) Introductory Product Data Sheet. In: Adhesives MT (ed.) UK

[19] Teroson MS 9399 (2020) Technical Data Sheet. In: Henkel (ed.)

[20] Weller B, Wünsch J (2013) Transparente Klebstoffe für Glas-Metall-Verbindungen. Stahlbau Spezial "Konstruktiver Glasbau" 82:169–183. 10.1002/stab.201390062

